# Single-Nucleus RNA-Seq Characterizes the Cell Types Along the Neuronal Lineage in the Adult Human Subependymal Zone and Reveals Reduced Oligodendrocyte Progenitor Abundance with Age

**DOI:** 10.1523/ENEURO.0246-23.2024

**Published:** 2024-03-01

**Authors:** Sofía Puvogel, Astrid Alsema, Hayley F. North, Maree J. Webster, Cynthia Shannon Weickert, Bart J. L. Eggen

**Affiliations:** ^1^Section Molecular Neurobiology, Department of Biomedical Sciences of Cells and Systems, University of Groningen, University Medical Center Groningen, Groningen 9700 AD, The Netherlands; ^2^Department of Human Genetics, Radboud University Medical Center, Donders Institute for Brain, Cognition and Behaviour, Nijmegen 6500 HB, The Netherlands; ^3^Schizophrenia Research Laboratory, Neuroscience Research Australia, Sydney, New South Wales 2031, Australia; ^4^School of Psychiatry, University of New South Wales, Sydney, New South Wales 2052, Australia; ^5^Laboratory of Brain Research, Stanley Medical Research Institute, Rockville 20850, Maryland; ^6^Department of Neuroscience and Physiology, Upstate Medical University, Syracuse, New York 13201

**Keywords:** neurogenesis, NSCs, SEZ, snRNAseq, SVZ

## Abstract

The subependymal zone (SEZ), also known as the subventricular zone (SVZ), constitutes a neurogenic niche that persists during postnatal life. In humans, the neurogenic potential of the SEZ declines after the first year of life. However, studies discovering markers of stem and progenitor cells highlight the neurogenic capacity of progenitors in the adult human SEZ, with increased neurogenic activity occurring under pathological conditions. In the present study, the complete cellular niche of the adult human SEZ was characterized by single-nucleus RNA sequencing, and compared between four youth (age 16–22) and four middle-aged adults (age 44–53). We identified 11 cellular clusters including clusters expressing marker genes for neural stem cells (NSCs), neuroblasts, immature neurons, and oligodendrocyte progenitor cells. The relative abundance of NSC and neuroblast clusters did not differ between the two age groups, indicating that the pool of SEZ NSCs does not decline in this age range. The relative abundance of oligodendrocyte progenitors and microglia decreased in middle-age, indicating that the cellular composition of human SEZ is remodeled between youth and adulthood. The expression of genes related to nervous system development was higher across different cell types, including NSCs, in youth as compared with middle-age. These transcriptional changes suggest ongoing central nervous system plasticity in the SEZ in youth, which declined in middle-age.

## Significance Statement

In the present study, single-nucleus sequencing analysis and immunostainings were performed to characterize the complete cellular niche of the adult human subependymal zone (SEZ), including youth and middle-aged donors. The authors identified most cell types found along the neuronal lineage, from neural stem cells (NSCs), neuroblasts, and immature and mature neurons, providing evidence of ongoing neurogenesis in the human SEZ neurogenic niche of youth and adults.

## Introduction

The subependymal zone (SEZ), also known as the subventricular zone (SVZ), lines the lateral walls of the lateral ventricles and constitutes a neurogenic niche that remains active during postnatal life (Doetsch and Alvarez-Buylla, 1996). In humans, neurogenesis remains high 1 year after birth and, although greatly attenuated, is sustained in mature adults, with evidence of proliferating cells and neuronal differentiation found in the adult human SEZ (Eriksson et al., 1998; Weickert et al., 2000; Curtis et al., 2007; van den Berge et al., 2010; Sanai et al., 2011; Ernst et al., 2014; Weissleder et al., 2017, 2021; Cipriani et al., 2018). Nonetheless, there is debate as to whether proliferative signals found in bulk tissue derive from neural stem cells (NSC) and/or neuronal progenitors (Dennis et al., 2016). The majority of NSCs in the adult human SEZ appear to exist in a predominantly quiescence state (Donega et al., 2022) and can be reactivated under certain conditions, governed by environmental and niche-derived factors (Curtis et al., 2003; Leonard et al., 2009; van den Berge et al., 2011; Gault and Szeler, 2021). Transcriptional markers for distinct stages of neurogenesis exist within the human SEZ throughout the entire life span (Weissleder et al., 2021). However, a single-cell/nucleus transcriptomic approach is necessary to establish whether all of the distinct cell types consistent with active neurogenesis can be identified and whether their proportions or transcriptional landscapes change from youth to maturity, when neuroplasticity generally becomes more restricted. Furthermore, if the cellular niche of adult NSCs modulates neurogenic potential, then a better understanding of the SEZ cellular heterogeneity in humans will provide clues as to what may influence neurogenic capacity.

In general, the fate of NSCs is modulated by the environment that surrounds them. Signals derived from the niche in the form of soluble factors or cell–cell contacts can induce NSCs to self-renew or give rise to neuroblasts, oligodendrocyte progenitor cells (OPCs), or immature astrocytes. For instance, angiogenesis and factors produced by endothelial cells like β-catenin and mitogenic growth factors increase NSC proliferation (Jin et al., 2002; Shen et al., 2004; Weissleder et al., 2016, 2019). Retinoic acid and cytokines can promote differentiation toward neurons or astrocytes, respectively (Környei et al., 2007; Borsini et al., 2015). Variations in the neurogenic capacity of the adult SEZ are observed under some pathological conditions that may disrupt or activate the SEZ. For example, PCNA-positive cells (a marker of cell proliferation) that costained with neuronal or astrocytic markers were increased in postmortem SEZ tissue of patients with Huntington's (Curtis et al., 2003), a neurodegenerative disease with severe neuropathology in the caudate nucleus. In Parkinson's patients, deep brain stimulation (DBS) near the SEZ increased the number of PCNA-positive cells that coexpressed δ-GFAP, an NSC marker, as compared with controls (Vedam-Mai et al., 2014). Similarly, ischemic injury and epilepsy increase the number of proliferative cells in the human SEZ (Macas et al., 2006; Kotagiri et al., 2014), while aging results in decreased expression of NSCs and cell cycle–related genes in the SEZ (Weissleder et al., 2021; Bitar et al., 2022). In schizophrenia, elevated inflammation is associated with altered neurogenic marker expression consistent with increased stem cell quiescence and reduced neurogenesis (North et al., 2021, 2022). The neurogenic potential of the adult human SEZ may be influenced by signals derived from different cell types, but the cellular diversity of this highly specialized region has not been resolved and is therefore the focus of this study.

Single-cell or nucleus RNA sequencing (sc/snRNAseq) enables transcriptional profiling at single-cell resolution (Tang et al., 2009) and provides information about the transcriptional heterogeneity of all the different cell types in a tissue. The mouse SEZ has been extensively studied with single-cell transcriptomics (Llorens-Bobadilla et al., 2015; Zywitza et al., 2018; Mizrak et al., 2019; Xie et al., 2020; Cebrian-Silla et al., 2021), but only one study has been published using snRNAseq of the adult human SEZ region. However, in their study of three aged individuals (mean age of 88), Donega et al. (2022) sampled the dorsal wall of the lateral ventricle adjacent to the corpus callosum, which is distinct from the primary neurogenic region found along the lateral wall of the lateral ventricle adjacent to the caudate nucleus. In fact, low levels of neurogenesis occur in the dorsal wall compared with the lateral wall and ventral regions of the human SEZ (Curtis et al., 2005). Additionally, the characteristics of neurogenic cells differ along the dorsal to ventral SEZ (Lim and Alvarez-Buylla, 2016), so the single-cell characterization of the lateral wall and ventral neurogenic zone was the focus of this study. Here, we used snRNAseq to reveal the transcriptional heterogeneity and relative abundance of different cell types that comprise the most actively neurogenic portion of the SEZ while comparing younger and older brains. Youth is generally associated with greater neuroplasticity and cognitive flexibility than later in life (Kupis et al., 2021), and while neurogenesis can contribute to plasticity, SEZ neurogenesis has not been fully characterized in youth and maturity. We therefore included four youth (average age of 20) and four middle-aged adults (average age of 50) to first characterize the cell types contributing to adult neurogenesis and secondly to evaluate whether the transcriptomic profile and/or cellular composition of the human SEZ differs with age. These data serve as a starting point to identify and characterize the transcriptional profiles and number of neurogenic cell types in the adult human SEZ, and to investigate the effect of age on this cellular niche.

## Materials and Methods

### Human brain tissue and nucleic isolation

**Figure 1.**
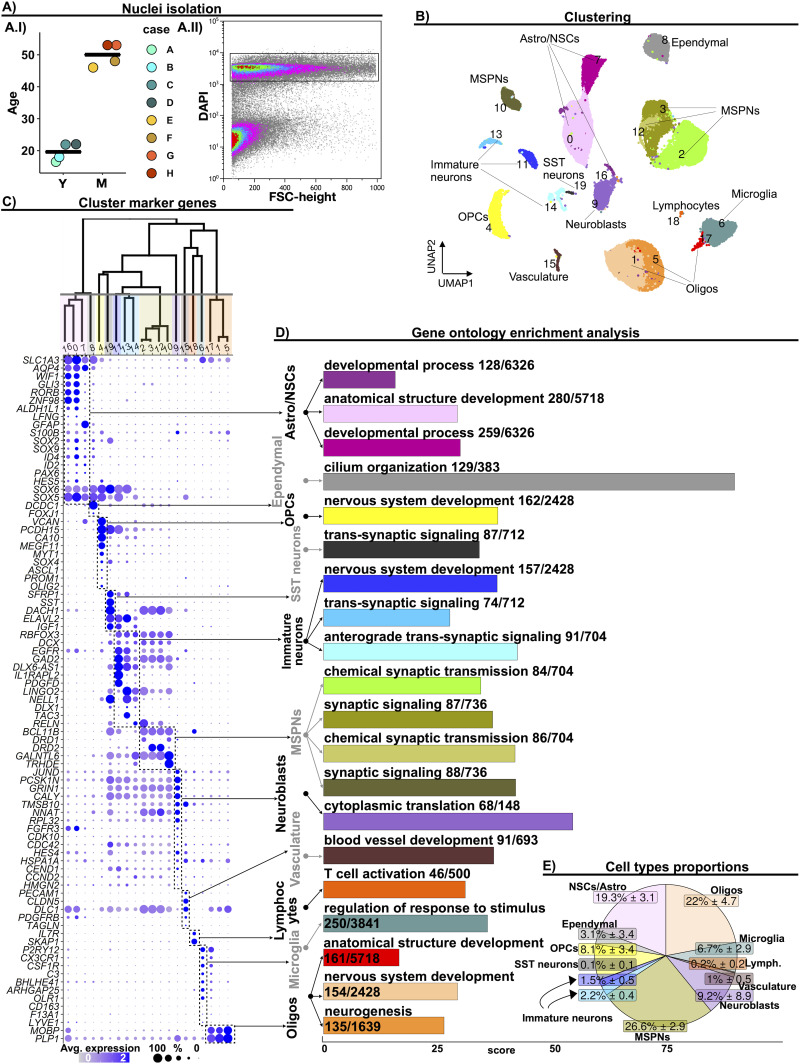
Cellular composition of the adult human SEZ. ***A***, Experimental workflow. ***A.I***, Scatter plot depicting the age of the donors, per age group. Each dot indicates a sample, and the horizontal line indicates the average age of the donors in the group. Youth (Y) and middle-aged (M) adults. ***A.II***, Fluorescence-activated nucleus sorting of DAPI^+^ events for snRNAseq. ***B***, UMAP depicting 36,626 nuclei from eight donors. The colors indicate nucleic clusters resulting from unsupervised clustering analysis of nucleic transcriptomic profiles. ***C***, Cluster marker genes. Top: dendrogram ordering the identified clusters based on hierarchical clustering. The horizontal gray line indicates the cutoff level used to group clusters with similar transcriptomic profiles. Grouped clusters were annotated as similar cell types. Bottom: cluster marker genes. The dot size indicates the fraction of nuclei expressing the gene, and the color depicts the gene scaled average expression. ***D***, Bar plot depicting the top enriched gene ontology term, for the more abundantly expressed genes, per cluster. Score: negative logarithm_10_ of the adjusted *p* value resulting from the enrichment analysis. ***E***, Pie chart depicting the average proportion of each cell type, considering the eight cases. Clusters were grouped based on the dendrogram depicted in (***C***) and annotated based on the expression of cellular-specific marker genes and gene ontology enrichment analysis. Neural stem cells (NSCs), astrocytes (Astro), oligodendrocytes (Oligos), lymphocytes (Lymph), medium spiny neurons (MSPNs), somatostatin (SST), oligoprogenitor cells (OPCs). Further supporting details for this figure are available in Extended Data Figure 1-1 and Extended Data Tables 1-1, 1-2, 1-3, and 1-4.

Subependymal zone (SEZ) samples from four youth (average age = 20) and four middle-aged adults (average age = 50) were obtained from the Stanley Medical Research Institute (SMRI; Extended Data Table 1-1). Postmortem brains were obtained from medical examiners with permission from the next-of-kin. Brain tissue samples were obtained from healthy individuals and showed no pathological abnormalities on histological examination. Ethical approval for the brain collection was through the Uniformed Services University for Health Sciences, Bethesda, Maryland. A cryostat was used to cut seven 100-µm-thick coronal sections from fresh frozen blocks of caudate nucleus. The SEZ tissue was dissected from each section, ∼2 mm deep to the lateral surface of the lateral ventricle adjacent to the caudate nucleus including the ventral regions (Extended Data Fig. 1-1). To ensure the quality of the brain tissue, we isolated RNA using an RNeasy Lipid Tissue Mini Kit (Qiagen, 74804) and measured RNA concentration and integrity on a Bioanalyzer 2100 (Aligent). The average RIN value of the samples was 8, and all presented a RIN > 7 (Extended Data Table 1-1). The SEZ tissues were lysed in a sucrose lysis buffer and the lysates were filtered through a 70 µm cell strainer. Then, nuclei were purified by ultracentrifugation through a dense sucrose buffer, as described in a study by Gerrits et al., (2021). After sucrose density centrifugation, ∼40,000 DAPI-positive events per sample were collected by FACS, and subjected to snRNAseq (Fig. 1*A*).

10.1523/ENEURO.0246-23.2024.f1-1 Figure 1-1 **Fresh-frozen tissue cutting and dissection of the SEZ.** **A)** Tissue from the rostral third of the basal ganglia was sectioned in the coronal plane into 7 × 100 μm sections. **B)** The SEZ (red dashed line) was dissected ∼2 mm deep to the surface of the lateral ventricle to include dorsal, middle and ventral regions. **C)** Luxol fast blue staining for myelin depicts the internal capsule separating the caudate nucleus and putamen as well as the external capsule surrounding the striatum. CN, caudate nucleus; IC, internal capsule; NAc, nucleus accumbens; SEZ, subependymal zone; Pu, putamen. Download Figure 1-1, TIFF file.

10.1523/ENEURO.0246-23.2024.t1-1 Extended Data table 1-1 Information about the cases and quality measurements of the SEZ samples obtained from them. Download Extended Data table 1-1, XLS file.

10.1523/ENEURO.0246-23.2024.t1-2 Extended Data table 1-2 Comparison of demographics between the age groups. Download Extended Data table 1-2, XLS file.

10.1523/ENEURO.0246-23.2024.t1-3 Extended Data table 1-3 Highly expressed genes per cluster. Download Extended Data table 1-3, XLS file.

10.1523/ENEURO.0246-23.2024.t1-4 Extended Data table 1-4 Quality measurements of the clusters. Download Extended Data table 1-4, XLS file.

### Demographics

The youth group ranged in age from 16 to 22 years (average = 20), and the middle-aged group ranged in age from 44 to 53 years (average = 50). Each group has one female and three males and has mixed ethnicity (3 Caucasian, 1 African American). The two age groups were matched for brain tissue quality variables (Extended Data Table 1-1). A parametric (Student's *t* test) or a nonparametric test (Mann–Whitney *U* test) was used, depending on the data distribution, to show the two age groups were not statistically different for brain pH, RNA integrity number (RIN), or postmortem interval (range from 7 to 40 h; Extended Data Table 1-2).

### Immunohistochemistry

Paraffin-embedded (10 µm), or fresh frozen (14 µm), coronal sections through the caudate nucleus were stained with polyclonal antibody to JUND (Thermo Fisher Scientific, #720035, 1:200), monoclonal antibody to PCSK1N (LifeSpan Biosciences, LS-C134062, 1:200) or polyclonal antibody to Olig2 (Abcam, ab42453, 1:400). Fresh frozen sections were thawed and fixed with 4% paraformaldehyde before the primary antibody was applied overnight 4°C. Paraffin sections were incubated in a 90°C bath of 0.01 M citrate buffer for 30 min before the primary antibody was applied overnight at 4°C. For 3,3’-diaminobenzide immunohistochemistry, following washes, sections were incubated in biotinylated secondary antibody (1:100), washed and incubated with avidin–biotin–peroxidase complex (Vectastain ABC kit, Vector Laboratories), treated with 3,3’-diaminobenzide, washed, stained with Nissl, and coverslipped. Olig2 immunofluorescence was performed on five youth (average age 18) and five middle-aged cases (average age 44) from a different cohort than the sequencing. After incubation in the primary antibody, sections were washed, incubated with secondary antibody (Alexa Fluor 594 Donkey Anti-Rabbit, Invitrogen, 1:500), washed, incubated with DAPI (1:1,000), treated with 5 mM CuSO4 in 50 mM AmmAc pH 5.0, and coverslipped with ProLong Gold Antifade Mountant (Thermo Fisher Scientific).

### snRNAseq library construction and sequencing

Single-nucleus cDNA libraries were constructed according to the user guide of Chromium Single Cell 3′ Reagents Kit v3.1 (10x Genomics). All samples were pooled in equimolar ratios and sequenced on a NextSeq 500 platform at the Research Sequencing Facility of the UMCG, Groningen, The Netherlands.

### snRNAseq data analysis

Sequencing reads were processed and aligned to the GRCh38 human genome using CellRanger v3.0.1 (Zheng et al., 2017). Filtered count matrices generated by Cell Ranger were loaded in R v4.0 with Seurat v4.0 (Hao et al., 2021). Nuclei with mitochondrial content > 5% were removed. Count information of the eight cases was log normalized and integrated according to guidelines for fast integration with reciprocal PCA (rPCA) in Seurat. Integrated data were scaled using the *ScaleData* function and regressing on “nCount_RNA,” “percent.mito,” “percent.ribo,” and “nFeature_RNA.” We did not regress on cell cycle–related genes because if there were differences in the expression of these genes between cell types or age groups, we were keen to identify them. Scrublet v0.2.1 was used to remove doublets. After these preprocessing steps, 36,626 nuclei were retained, with 4,676 reads per nuclei on average. Unbiased clustering analysis (dim = 30, k.parameter = 10, res = 0.4) followed by examination of marker gene expression was performed to identify all major cell types. The *FindAllMarkers* function from Seurat v4.0 with default parameters was used to identify differentially expressed genes per cluster (marker genes). Marker genes per cluster are provided in Extended Data Table 1-3. Quality measurements of the clusters are provided in Extended Data Table 1-4.

#### Hierarchical clustering

A Person's correlation matrix was calculated on the average transcriptomic profiles of the clusters, considering only highly variable mRNAs. The correlation matrix was used as an argument for the *heatmaply* function of Heatmaply R package v1.3.0 (Galili et al., 2018), specifying hclust_method = “single.”

#### Gene ontology enrichment analysis

Gene ontology (GO) enrichment analysis was performed on the abundantly expressed genes of each cluster (log_2_FC > 0.5 and adjusted *p* value < 0.05), using the *gost* function of the R package gprofiler2 v0.2.1. *p* values were adjusted for multiple-comparisons setting correction_method = g_SCS. Redundancy of enriched biological process GO terms was accounted for with clustering analysis and aggregating terms with high semantic similarity using the functions *calculateSimMatrix*, setting ont = “BP” and *reduceSimMatrix* with threshold = 0.7 of the rrvgo v1.2.0 R package.

#### Characterization of clusters expressing neuronal and neural stem cell marker genes

Clusters expressing neuronal and/or NSC marker genes (clusters 16, 0, 7, 8, 19, 11, 13, 14, 2, 3, 12, 10, and 9) were extracted from the dataset. Count information of these nuclei was reintegrated across the samples using canonical correlation-based integration. Downstream pseudotime and enrichment analyses, detailed below, were performed on this nucleic subset.

##### Pseudotime analysis

A pseudotime trajectory was calculated through the transcriptomic profiles of the nucleic subset, following the guidelines of the R package Destiny v3.4.0 (Angerer et al., 2016). Briefly, count data of highly variable transcripts within the nucleic subset were used to identify the diffusion components with *DiffusionMap*, using default parameters. The *DPT* function was used to calculate the pseudotime trajectory, and the *random_root* parameter was activated to automatically identify the starting of the trajectory. To identify the most relevant driver genes of the pseudotime trajectory, we used *gene_relevance* (Angerer et al., 2020) with dims = 1:3. Additional trajectory inference analysis including every nucleus, from all clusters, was performed using the R package Monocle3 v1.3.4 (Trapnell et al., 2014). For this purpose, we generated a csd object with the *new_cell_data_set* function, using the count and meta data stored in the Seurat object (including the UMAP information and cluster assignation for every nucleus). Then, the *learn_graph*, setting close_loop = false, and *order_cells* functions were used to infer the pseudotime, setting the parameter root_pr_nodes to nuclei belonging to clusters 7, 0, and 16, as these may constitute the neural stem cells. We repeated this analysis for youth and middle-aged nuclei, independently.

##### Enrichment of marker genes of mouse SEZ cell types

We used the *AddModuleScore* function of Seurat, with default parameters, to evaluate the enrichment of gene sets associated with mouse SEZ cell types in our human SEZ clusters.

##### Enrichment of neuropsychiatric diseases associated genes

The R package MAGMA.Celltyping v2.0.1 with linear enrichment mode (de Leeuw et al., 2015; Skene et al., 2018) was used in R v4.1.2 to evaluate whether genes previously associated with different neurodevelopmental diseases were specifically expressed by any SEZ neuronal and/or NSC cluster. Log normalized data of the variable genes within the nucleic subset were used to generate a CellTypeData (CTD) file, with the *generate_celltype_data* function of EWCE v1.3.3 (Skene and Grant, 2016). *load_rdata* of EWCE was used to load the generated CTD in R. The genes associated with neurodevelopmental diseases were obtained from genome-wide association studies (GWAS) for schizophrenia (Schizophrenia Working Group of the Psychiatric Genomics Consortium, 2014), bipolar disorder (Howrigan et al., 2023), and autism (Grove et al., 2019). The summary statistics were downloaded from the IEU open GWAS Project database (Ben et al., 2020), and the *format_sumstats* function from MungeSumstats v1.6.0 (Lyon et al., 2021; Murphy et al., 2021) was used to standardize the files. The *map_snps_to_genes* of MAGMA.Celltyping was then used to annotate SNPs onto their neighboring genes. The files generated with *map_snps_to_genes* were used for the *celltype_associations_pipeline* of MAGMA.Celltyping, to estimate the enrichment of genes associated with the mentioned diseases in our subset of neuronal and NSC clusters, setting ctd_species = “human” and run_linear = true.

#### Group comparisons

We compared the age groups in terms of the transcriptomic profiles and relative abundance of the different clusters.

##### Identification of differentially expressed genes between youth and middle-age

To test for differences in the transcriptomic profiles of the SEZ clusters between youth and middle-age, we made a prior selection of genes per cluster that met the following conditions: (1) expressed in at least 25% of the nuclei in one of the two age groups and (2) with absolute log_2_FC > 0.25 between the two groups. Only expression data derived from samples that contributed more than three nuclei to the given cluster were considered. The *zlm* function of the R package MAST v1.16.0 (Finak et al., 2015) was used to identify the differentially expressed genes (DEGs) between youth and middle-age in each cluster. We corrected gene expression by the cellular detection rate and included a random intercept per case to account for donor-related structure in the data. The results of this analysis are provided in Extended Data Table 3-1. After correction, genes were considered differentially expressed when the effect of age had a log_2_FC > 0.1, with a false discovery rate (FDR) adjusted *p* value < 0.05.

##### Comparison of cluster proportions between youth and middle-age

A generalized linear model (GLM) was used to test if age (youth or middle-age) affects the probability of a nucleus belonging to a given cluster. We used the *glmer* function of the lme4 R package v1.1.27.1 (Bates et al., 2015) with a quasibinomial distribution, because of the binary nature of the response variable (the nucleus either belongs to the given cluster or not). Considering that a model was created per cluster, the obtained *p* values were corrected with the Bonferroni method, and the number of comparisons was set to the total number of clusters.

##### Estimation of cluster proportions in bulk transcriptomic data

The “Impute cell fractions” method of CIBERSORTx software (Newman et al., 2019) was used to estimate the proportion of our SEZ snRNAseq clusters in bulk transcriptomic data from SEZ samples of 20 donors, aged from 15 to 86 years old (Bitar et al., 2022). These donors were previously assigned to four different age groups, adolescents (average age = 15.5 years), young adults (average age = 23 years), adults (average age = 42.3 years), and aging (average age = 86 years; Bitar et al., 2022). The signature matrix, reflecting the average transcriptomic profiles per SEZ snRNAseq cluster, was generated using count data of the highly variable transcripts. The mixture file was generated with the bulk RNA sequencing data (Bitar et al., 2022). Counts of each expressed gene were divided by the total number of counts in each sample and multiplied by 1,000,000 (CPM normalization). To corroborate the differences in the relative abundance of SEZ clusters associated with age, identified in the snRNAseq dataset, we compared the estimated proportion in the bulk RNA sequencing data, obtained with CIBERSORTx, of cluster 4 (oligo progenitors), cluster 6 (microglia), and cluster 13 (neuronal cluster) between the four different age groups. We used a Kruskal–Wallis test and post hoc analysis was performed with the Dunn test. *p* values were FDR corrected.

##### Validation of changes in differentially expressed genes and oligo progenitors, microglia, and immature neurons cluster 13 markers using bulk transcriptomic data

The bulk transcriptomic data of the SEZ (Bitar et al., 2022) was also used to validate the identified DEGs and changes in the expression of marker genes of cluster 4 (oligo progenitors), cluster 6 (microglia), and cluster 13 (neuronal cluster) associated with age. For this purpose, we calculated Spearman correlations between gene expression and donor age for DEGs in each cell type and marker genes specific to microglia, OPCs, and the immature neuron cluster 13.

##### Validation of changes in oligo progenitors, microglia, and immature neuron cluster 13 marker genes using qRT-PCR

In an independent cohort of brain tissue from 19 youth (aged 15–25 years, mean age 20) and 19 middle-aged adults (aged 36–62, mean age 48), we dissected the SEZ, extracted RNA, and synthesized cDNA for qRT-PCR using the same methods detailed in Weissleder et al. (2021). The youth and middle-age groups were matched for demographic and postmortem factors such as sex, postmortem interval, RNA integrity number (RIN), and brain pH (all *p* > 0.55). Gene expression was measured for markers of oligo progenitors (*PROM1*), microglia (*C3*), and immature neurons (*DLX1*) using the BioMark HD system (Fluidigm). The following Taqman Assay IDs were used: *PROM1*, Hs01009259_m1; *C3*, Hs00163811_m1; and *DLX1*, Hs00698288_m1 (Thermo Fisher Scientific). The “no template controls” did not produce a signal. Gene expression was normalized to the geometric mean of three housekeeper genes *ACTB*, *GAPDH*, and *TBP*, which were chosen based on stable expression across age groups (all *p* > 0.24, data not shown). The geometric mean did not significantly differ between age groups (*p* = 0.40, data not shown). Outliers were defined as values greater than two standard deviations from the group mean and were removed from the analysis. Shapiro–Wilk tests were used to determine the normal distribution, and Mann–Whitney *U* tests were performed for genes not meeting normality. Levene's tests were used to test the equality of variances prior to independent sample *T*-tests for genes with normal distribution.

#### Code accessibility

The code used for snRNAseq analysis is available at https://github.com/sofiapuvogelvittini/humanSEZ_snRNAseq_analysis. Analyses were conducted on a 96-cluster running with the Ubuntu operating system, facilitated by the Molecular Neurobiology section of the UMCG.

## Results

### Single-nucleus RNA sequencing identifies major neurogenic cell types in the adult human SEZ

The snRNAseq data from both age groups were pooled and analyzed together. Unbiased cluster analysis of the transcriptomic profiles of 36,626 nuclei revealed 20 clusters of cell types within the lateral wall and ventral floor of the lateral ventricle (Fig. 1*B*). To identify the major cell types, clusters with similar transcriptomic profiles were grouped together, based on hierarchical clustering analysis (Fig. 1*C*, top) and annotated into 11 different main cell types, based on the expression of cell type-specific marker genes (Fig. 1*C*, bottom) and gene ontology enrichment analysis (Fig. 1*D*).

The nuclei in cluster 8 were annotated as ependymal, because of their high expression of doublecortin domain–containing protein 1 (*DCDC1*) and *FOXJ1*, both marker genes of ependymal cells (Ortiz-Álvarez et al., 2019). Ependymal nuclei corresponded to 3.1% of cell types in the SEZ (Fig. 1*E*). Interestingly, the ependymal cluster also highly expressed the stem cell marker genes *ID4*, *SOX2*, and *SOX9* (Scott et al., 2010).

Clusters 16, 0, and 7 were annotated as Astro/NSCs, together representing 19.3% of the cell types (Fig. 1*E*). Clusters 16, 0, and 7 (Fig. 1*C*) were enriched for genes expressed by neuronal progenitor or NSCs, such as *ID4* (Blomfield et al., 2019; Zhang et al., 2019), *SOX2* (Episkopou, 2005), and *SOX5* (Azim et al., 2009; Quiroga et al., 2015), as well as astrocyte marker genes, such as *SLC1A3* and *AQP4* (Batiuk et al., 2020). Cluster 0 highly expressed *HES5*, involved in the maintenance of the undifferentiated state in neuronal progenitor cells (Manning et al., 2019), while both clusters 0 and 16 abundantly expressed *GLI3* and *LFNG*, which maintain neural progenitors actively in the cell cycle (Wang et al., 2011) and regulate NSC cycling (Semerci et al., 2017). Cluster 0 and 7 highly expressed *PAX6*, which modulates NSC differentiation (Kallur et al., 2008; Sansom et al., 2009). In each of these three clusters, the most enriched gene ontology biological term was associated with development (Fig. 1*D*).

Cluster 4 was annotated as OPCs, based on the expression of OPC marker genes such as *OLIG2*, *VCAN*, *PCDH15*, *CSPG4*, *MEGF11*, and *PROM1* (Sim et al., 2002; Grubman et al., 2019; Fig. 1*C*; Extended Data Table 1-3), and represented 8.1% of the cell types.

Cluster 9 was annotated as neuroblasts and represented 9.2% of the cell types in human SEZ. Cluster 9 expressed neuronal genes, such as NMDA glutamate receptor subunit 1 (*GRIN1*) and the gene coding for Calycon (*CALY*; Kruusmägi et al., 2007); however, it did not express *RBFOX3* (NeuN), which is typically expressed by postmitotic neurons (Dredge and Jensen, 2011). Nuclei in cluster 9 abundantly expressed genes that were previously identified as highly expressed by neuroblasts in the adult mouse and primate hippocampal neurogenic niches, such as *TMSB10*, *NNAT*, and *HMGN2* (Artegiani et al., 2017; Hao et al., 2022). These cells expressed markers for stem cell activation (*RPL32*; Dulken et al., 2017), and genes that regulate the cell cycle, proliferation, and early neuronal differentiation (*CCND2*, *CDK10*, *CDC42*, *FGFR3*, *CEND1*; Kowalczyk et al., 2004; Katsimpardi et al., 2008; Vadodaria et al., 2013; Yeh et al., 2013). The top enriched term in cluster 9 abundantly expressed genes was a cytoplasmic translation (Fig. 1*D*). Mouse SEZ neuroblasts and active NSCs (aNSCs) highly express ribosomal and translation-related genes too (Llorens-Bobadilla et al., 2015; Xie et al., 2020), suggesting that cluster 9 may share features with mouse neuroblasts and/or aNSCs.

Clusters 11, 13, and 14 were annotated as immature neurons and together represented 3.7% of the identified cell types in the human SEZ (Fig. 1*E*). Although clusters 11, 13, and 14 exhibited high *RBFOX3* expression, they also abundantly expressed *DLX6-AS1*, which is expressed in neuronal progenitor cells (Dulken et al., 2017), as well as in inhibitory neurons (Wang et al., 2018; Nagy et al., 2020; Tran et al., 2021). They all expressed *DCX*, a marker of immature neurons that is associated with their differentiation and migration (Ayanlaja et al., 2017). Clusters 13 and 14 expressed *DLX1*, another important gene for neuronal differentiation (Gotz et al., 2016). Additionally, clusters 11 and 13 highly expressed *EGFR*, also expressed by neuronal progenitors and relevant for their proliferation and survival (Weickert and Blum, 1995; Cochard et al., 2021). Cluster 11, the cluster with the highest *DLX6-AS1* expression activity (Extended Data Table 1-3), also highly expressed *PDGFD*, which is expressed by human radial glial cells in the ventricular zone during corticogenesis (Lui et al., 2014).

We identified five clusters of mature neurons (19, 2, 3, 12, and 10). Cluster 19 was annotated as somatostatinergic neurons (SST neurons), based on high *SST* expression levels. SST neurons expressed *GAD2* but to a lower extent than the rest of the neuronal clusters in the SEZ. All the other neuronal clusters highly expressed *GAD2*, suggesting that these nuclei derived from GABAergic inhibitory neurons. We identified four clusters of medium spiny neurons (clusters 2, 3, 12, and 10; MSPNs; 26.6% of the cell types), which probably were derived from the adjacent striatum, based on their high expression of *BCL11B*, *DRD1*, or *DRD2* (Arlotta et al., 2008; Zywitza et al., 2018).

Clusters 17, 1, and 5 highly expressed the myelin-associated oligodendrocyte basic protein (*MOBP*) gene and were identified as oligodendrocytes, likely derived from the adjacent white matter. Together, the three oligodendrocyte clusters represented 22% of the cell types.

Cluster 6 contained microglia nuclei, enriched for *P2RY12*, *CX3CR1*, and *C3* (Galatro et al., 2017; Alsema et al., 2020; Gerrits et al., 2021). Microglia accounted for 6.7% of the cell types. Nuclei of cluster 15 were annotated as vascular cells and represented 1% of the cell types (Fig. 1*E*), while cluster 18 corresponded to lymphocyte nuclei (0.2% of the cell types) that abundantly expressed *IL7R* and *SKAP1* (Smith et al., 2016; Gerrits et al., 2021).

### Adult human cell types in the SEZ overlap with mouse neurogenic cells and localize on a pseudotime/differentiation trajectory driven by *CALM1* and *FRMPD4*

**Figure 2.**
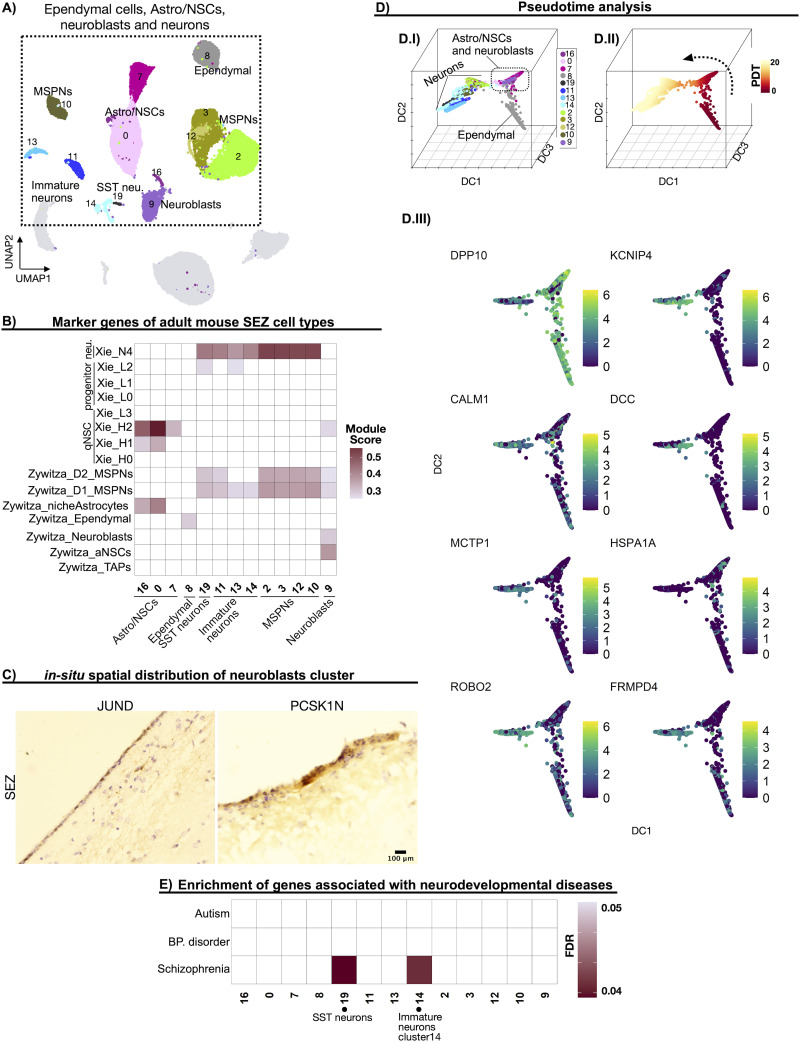
Profiling of SEZ clusters expressing NSCs and neuronal marker genes. ***A***, UMAP highlighting ependymal, Astro/NSC, neuroblast, and neuronal nuclei, which were extracted and further characterized. ***B***, Heatmap depicting average module scores of gene sets associated with mouse SEZ cell types (Zywitza et al., 2018; Xie et al., 2020). ***C***, Representative IMH of JUND and PCSK1N in paraffin-embedded and fresh adult human SEZ sections, respectively. ***D***, Pseudotime analysis. ***D.I***, Nuclei are ordered in a three-dimensional diffusion map (DC, diffusion components). The colors indicate the original nucleic clusters. ***D.II***, Pseudotime (PDT) estimation, indicated in a color code, is calculated based on the diffusion components. The origin of the pseudotime trajectory was automatically identified. The pseudotime trajectory reveals a progression from the ependymal to the neuronal nuclei, going through the Astro/NSC and neuroblast nuclei. ***D.III***, Two-dimensional diffusion maps of the most relevant genes driving the pseudotime depicted in ***D.II***. The color of the dot indicates the gene expression level in each nucleus. ***E***, Heatmap depicting the association of gene sets related to different neurodevelopmental diseases with our Astro/NSC, neuroblast, ependymal, and neuronal clusters. The color code indicates the FDR corrected *p* value obtained from associating the particular gene set with each cluster. Further supporting details for this figure are available in Extended Data Figures 2-1 and 2-2.

To further characterize the cell types expressing NSC and/or neuronal genes, we extracted ependymal cells, Astro/NSCs, neuroblasts, immature neurons, SST neurons, and MSPNs (Fig. 2*A*) from the dataset and subjected them to enrichment and pseudotime analyses.

10.1523/ENEURO.0246-23.2024.f2-1 Figure 2-1 **Additional pseudotime analysis with Monocle3.** **A)** Trajectory graph inferred with Monocle3, including all cell types. The numbers signify potential origins of pseudotime trajectories. **B)** Pseudotime analysis including all cell types. **C)** Pseudotime analysis in youth (Y) nuclei, including all cell types. **D)** Pseudotime analysis in middle-age (M) nuclei, including all cell types. Root nuclei were selected in clusters 7 and 0 and 16, as these may constitute the neural stem cell populations. The numbers in B-D denote the selected nuclei serving as the initial points for pseudotime trajectories. Download Figure 2-1, TIF file.

10.1523/ENEURO.0246-23.2024.f2-2 Figure 2-2 **Additional pseudotime analysis with Destiny.** Trajectory graph inferred with Destiny, including all cell types and nuclei. **A)** Nuclei are ordered in a three-dimensional diffusion map (DC=diffusion components). Colors indicate original nuclei clusters. A.I-A.II) Present the identical graphs from different perspectives. **B)** Pseudotime (PDT) estimation, indicated in a color code, calculated based on the diffusion components. The origin of the pseudotime trajectory was automatically identified. OPCs situated in proximity to immature neurons, consistent with findings from Monocle3 analysis. Download Figure 2-2, TIF file.

Considering that mouse SEZ has been characterized to a greater extent at the single-cell level and humans and mice share some neurogenic features (Lazarov and Marr, 2013), we evaluated the expression of adult mouse SEZ progenitor and neuronal marker genes (Zywitza et al., 2018; Xie et al., 2020) in the subset of our human NSC/neuronal SEZ clusters. The human Astro/NSC clusters were enriched in mouse marker genes for niche astrocytes, and H1 and H2 quiescent neural stem cells (qNSCs; Fig. 2*B*; Xie et al., 2020). Indicating that astrocytes in the adult human SEZ may maintain NSC properties, as was previously suggested (Sanai et al., 2004).

The human neuroblast cluster (cluster 9) was enriched in genes highly expressed by mouse neuroblasts and active neural stem cells (aNSCs; Zywitza et al., 2018). To identify the spatial distribution of this cell type in the human SEZ, we stained for neuroblast markers JUND (Meixner et al., 2004; Millena et al., 2016; Elliott et al., 2019) and PCSK1N (Morgan et al., 2010). JUND and PCSK1N-positive cells showed a similar localization pattern in the human SEZ, with most labeled cells identified along the ventricular edge of the SEZ where neuroblasts are known to migrate (Doetsch et al., 1997), with occasional cells that appear away from the ependymal layer (Fig. 2*C*).

None of the human SEZ clusters were enriched for marker genes of mouse SEZ transient amplifiers (TAPs; Fig. 2*B*) or for marker genes of mouse “L clusters” (Zywitza et al., 2018; Xie et al., 2020), which were described as the mitotically most active cell types in the mouse SEZ. SST neurons (cluster 19), immature neurons (clusters 11, 13, and 14), and MSPNs (clusters 2, 3, 12, 10) neuronal clusters were enriched for genes abundantly expressed by cluster N4 “mouse SEZ neurons” (Xie et al., 2020; Fig. 2*B*). Moreover, our human MSPNs were enriched for genes highly expressed by the two MSPNs clusters detected in mouse SEZ by Zywitza et al. (2018).

We calculated a pseudotime trajectory based on the transition probability through the subset of nuclei expressing neuronal and/or NSC marker genes, estimated with a random diffusion model (Fig. 2*D.I*; Angerer et al., 2016). We used an automatized method that identifies the most distant nucleus indicating the root cell type of the pseudotime, based on the diffusion model, from randomly selected nuclei in the dataset. The algorithm identified ependymal nuclei as the origin of the pseudotime trajectory, which advanced toward the neuronal nuclei through the Astro/NSC and neuroblast nuclei (Fig. 2*D.II*). These observations further support a progenitor cell profile associated with Astro/NSC and neuroblast nuclei and suggest that adult ependymal and NSCs in the ventricular subventricular zone are sister cell types (Ortiz-Álvarez et al., 2019). To identify the genes that potentially led the differentiation transitions, we used a recently published methodology to identify the driver genes of nonlinear pseudotime trajectories (Angerer et al., 2020). The most relevant genes, drivers of the pseudotime, are indicated in Figure 2*D.III*. The expression of *CALM1* and *FRMPD4* increased along with the pseudotime, while *DPP10* and *HSPA1A* expression increased from ependymal nuclei to Astro/NSCs reaching the highest expression activity in neuroblasts and then decreased toward neuronal nuclei. Conversely, the expression activity of *KCNIP4*, *ROBO2*, *DCC*, and *MCTP1* varied locally within the subset of neuronal nuclei; therefore, these genes may constitute drivers of the maturation of distinct types of neurons. To complement and extend the pseudotime analysis, we inferred a pseudotime trajectory using an alternative tool, Monocle3, including all cell types in the SEZ (Extended Data Fig. 2-1A,B). Considering our previous analysis, where clusters 7, 0, and 16 were identified as potential NCSs, we designated nuclei within these clusters as the starting points of the trajectory. The pseudotime trajectory exhibited some bifurcations, revealing two distinct paths originating from the Astro/NSCs and leading toward the neuronal nuclei, passing through neuroblasts. Notably, one of these trajectories (Extended Data Fig. 2-1B, highlighted with a dotted arrow) began with the Astro/NSCs, progressed to the neuroblasts, and then transitioned toward immature neuronal clusters, providing further evidence of progenitor features for the neuroblast cluster. Interestingly, according to the pseudotime trajectory, OPCs were found to be closer to immature neurons than to mature oligodendrocytes. This could be attributed to the high expression of progenitor-related marker genes in both OPCs and immature neurons, causing them to cluster closely together. The same result was further corroborated with Destiny pseudotime analysis, including all cell types and nuclei (Extended Data Fig. 2-2). To explore potential age-specific trajectories, we conducted Monocle3 pseudotime analysis separately for youth and middle-aged nuclei (Extended Data Fig. 2-1C,D). In this context, the pseudotime trajectories followed the same path as the complete dataset, with no differences between the two age groups.

Next, we tested whether the human SEZ clusters expressing neuronal or NSC marker genes were enriched for genes associated with neuropsychiatric disorders that also have reported alterations in the SEZ, such as schizophrenia (Al-Shammari et al., 2018; Weissleder et al., 2021), bipolar disorder (Weissleder et al., 2021), and autism (Wegiel et al., 2010; Kotagiri et al., 2014). Two neuronal clusters were enriched for genes associated with schizophrenia (Fig. 2*E*), the SST neurons and immature neurons that also abundantly expressed *RELN* (cluster 14, Fig. 1*C*). We did not detect genes associated with bipolar disorder or autism enriched for any SEZ neuronal or NSC clusters.

### The transcriptomic profile of neuronal and nonneuronal cell types changes between youth and middle adulthood

**Figure 3.**
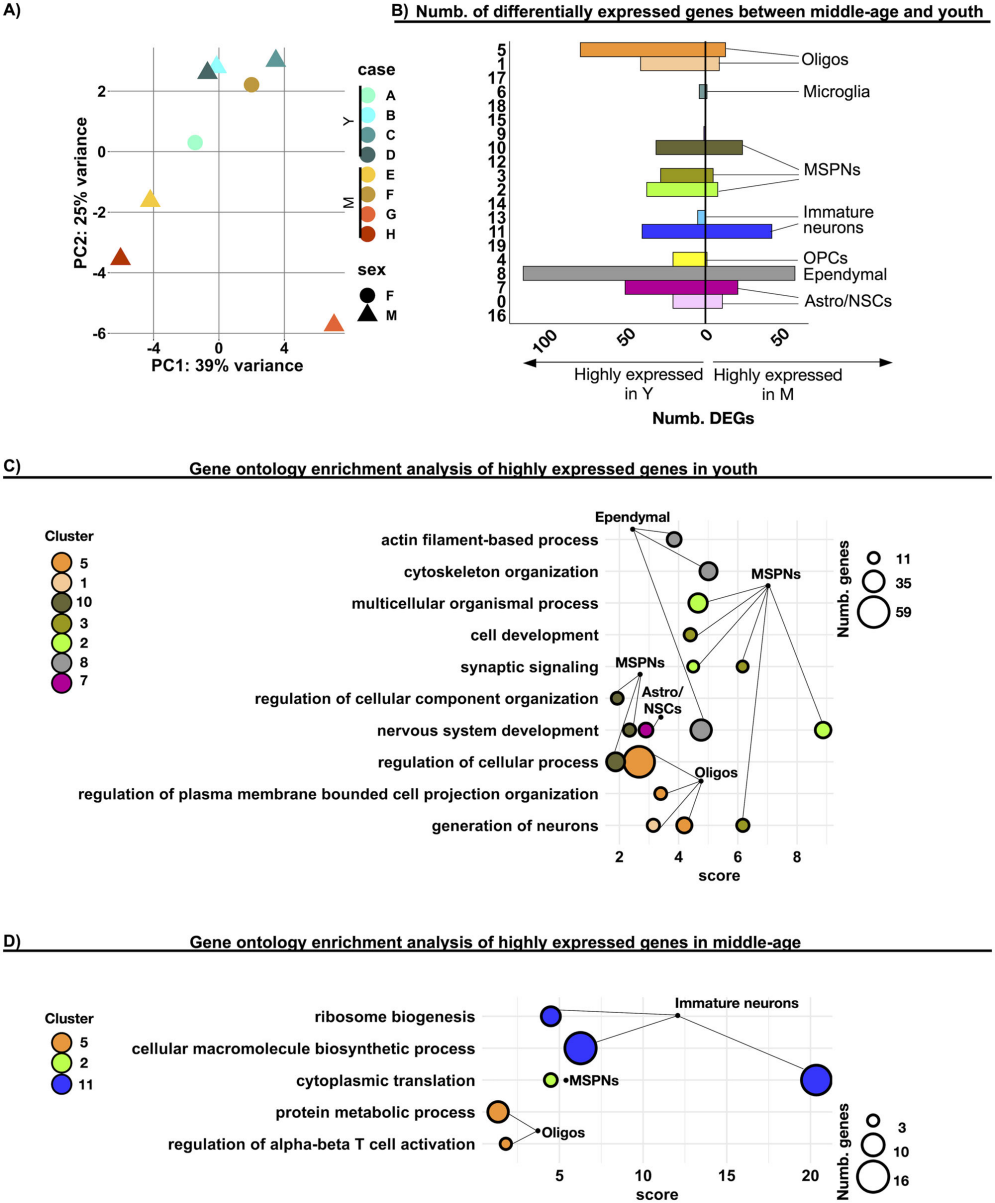
Differences in the transcriptomic profiles of cell types in the SEZ between middle-age and youth. ***A***, Principal component analysis of pseudobulked samples. Each symbol indicates a donor and its shape donor sex. Youth (Y) and middle-aged adults (M). ***B***, Bar plot depicting the number of identified differentially expressed genes (DEGs) between M and Y (absolute log_2_FC > 0.25 and FDR adjusted *p* value < 0.05, detailed in Materials and Methods), across the different clusters. ***C***, Scatter plot depicting the top three significantly enriched terms in the highly expressed genes in youth, across the different clusters. The color of the dot indicates the cluster, and its size indicates the number of intersected genes between the list of DEGs and the genes associated with the particular term. Lines/edges connect clusters corresponding to the same cell type, defined with hierarchical clustering analysis in Figure 1*E*. Score: negative logarithm_10_ of the adjusted *p* value resulting from the enrichment analysis. ***D***, Scatter plot depicting the top three significantly enriched terms in the highly expressed genes in middle-age. Further supporting details for this figure are available in Extended Data Figures 3-1, 3-2, and 3-3 and Extended Data Tables 3-1, 3-2 and 3-3.

Having identified the cellular identity of all the different SEZ clusters, we then tested for potential differences in the transcriptomic profiles between youth and middle-age. Principal component analysis (PCA; Fig. 3*A*) showed that the samples were segregated based on donor age. We identified 569 differentially expressed genes (DEGs; log_2_FC > 0.25; adjusted *p* value < 0.05) within various cell type clusters when comparing youth and middle-age SEZ cells (Fig. 3*B*; Extended Data Figs. 3-1 and 3-2; Extended Data Table 3-1). Across different cell types, some genes, such as *ANO4*, *PBX3*, *MAN2A1*, *FLRT2*, *GABRA2*, *CPE*, *RAB3C*, *SEMA6D*, *ADGRL3*, *CNTN4*, and *HS3ST5*, were depleted in middle-age samples, while *SLC6A1-AS1* and *ITPKB* were enriched in middle-age samples (Extended Data Table 3-2). This suggests a generalized effect of age on the expression of these transcripts across different cell types.

10.1523/ENEURO.0246-23.2024.f3-1 Figure 3-1 **Differentially expressed genes between youth and middle-age in non-neuronal clusters.** Heatmaps depicting scaled expression level of the differentially expressed genes between youth and middle-age (absolute log_2_FC > 0.25 and FDR adjusted *p* value < 0.05, detailed in Methods) in **A)** Oligodendrocytes cluster 1, **B)** Oligodendrocytes cluster 5, **C)** Ependymal cells cluster 8 and **D)** Astro/NSCs cluster 7. Download Figure 3-1, TIF file.

10.1523/ENEURO.0246-23.2024.f3-2 Figure 3-2 **Differentially expressed genes between youth and middle-age in neuronal clusters.** Heatmaps depicting scaled expression level of the differentially expressed genes between youth and middle-age (absolute log_2_FC > 0.25 and FDR adjusted *p* value < 0.05, detailed in Methods) in **A)** MSPNS cluster 2, **B)** MSPNs cluster 3, **C)** MSPNs cluster 10 and **D)** immature neurons of cluster 11. Download Figure 3-2, TIF file.

10.1523/ENEURO.0246-23.2024.f3-3 Figure 3-3 **Validation of differentially expressed genes with bulk transcriptomic data.** **A)** Heatmap depicting differentially expressed genes between youth and middle-age for which its expression activity was also significantly correlated with age in a bulk RNA sequencing dataset of the SEZ, collected from 20 donors spanning an age range of 15 to 86 years. The correlation between gene expression activity and age is shown in color code, for genes that shown a consistent change associated with age in both the snRNA and the bulk sequencing dataset. Corresponding *p* values for each correlation are provided. **B-C)** Scatter plots showing normalized expression activity in relation to age, for genes with possitive and negative correlation with age, respectively. Download Figure 3-3, TIF file.

10.1523/ENEURO.0246-23.2024.t3-1 Extended Data table 3-1 Differentially expressed genes in middle-age as compared to youth in each cluster. Download Extended Data table 3-1, XLS file.

10.1523/ENEURO.0246-23.2024.t3-2 Extended Data table 3-2 Genes with different expression level between middle-age and youth across multiple cell types. Download Extended Data table 3-2, XLS file.

10.1523/ENEURO.0246-23.2024.t3-3 Extended Data table 3-3 Spearman correlations between gene expression and age in a bulk RNA sequencing dataset of the human SEZ, for differentially expressed genes identified in the snRNAseq data. Download Extended Data table 3-3, XLS file.

Both nonneuronal (Extended Data Fig. 3-1) and neuronal clusters (Extended Data Fig. 3-2) contained DEGs between youth and middle-age. Ependymal cells had the highest number of DEGs between the two age groups (Fig. 3*B*; 176 DEGs). Gene ontology enrichment analysis indicated that genes highly expressed in young ependymal cells, such as *SHROOM3*, *SOX9*, *EFNA5*, *S100A10*, *FGFR2*, and *VIM*, were enriched for “actin filament-based process,” “cytoskeleton organization,” and “nervous system development” (Fig. 3*C*). The highly expressed genes in young Astro/NSC cluster 7, including *CECR2*, *CDH4*, *PTPRM*, *SEMA6D*, and *SEMA5A*, were also enriched for the gene ontology term “nervous system development.”

Oligodendrocytes of cluster 5 exhibited the second highest number of DEGs between youth and middle-age. The genes more abundantly expressed in young oligodendrocytes (cluster 5), such as *HECW2*, *DISC1*, *FUT9*, *TNIK*, and *MACF*, were enriched in “regulation of cellular process” and “generation of neurons” (Fig. 3*C*). In contrast, the genes enriched in older oligodendrocytes, such as *B2M*, *ITPKB*, *SH3RF1*, and *CD81*, were related to inflammation: “regulation of alpha–beta T-cell activation” (Fig. 3*D*), in line with previous studies showing a potential immunomodulatory role of oligodendrocytes (Falcão et al., 2018).

For neuronal clusters (Extended Data Fig. 3-2), the MSPNs nuclei exhibited higher expression of the *RELN*, *SYT4*, *CNTN4*, *FLRT2*, *NPAS2*, *NREP*, *PTPRD*, *SLIT2*, *IL1RAPL1*, and *HDAC9* genes in youth. These young MSPNs genes related to “nervous system development” and “generation of neurons” (Fig. 3*C*). In contrast, immature neuron cluster 11 exhibited higher expression of genes such as *DHX36*, *EIF4A2*, and other genes coding for ribosomal proteins (*RP*), related to “cytoplasmic translation,” “cellular macromolecule biosynthetic process,” and “ribosome biogenesis” in middle-age. The highly expressed genes in middle-age MSPNs of cluster 2 were also related to “cytoplasmic translation” and included genes coding for different *RP* genes (Fig. 3*D*).

To validate some of the identified DEGs, we examined their correlation with donor age using a previously published bulk RNA sequencing dataset derived from human SEZ samples. This dataset encompassed samples collected from 20 donors spanning an age range of 15–86 years. In this analysis, we identified 44 genes in the bulk dataset that exhibited the same directional change in expression associated with age as we observed in the snRNAseq data, including genes such as *DISC1*, *NREP*, and *CDH4* (Extended Data Fig. 3-3, Extended Data Table 3-3), among others. For the genes that did not confirm their correlation in the bulk RNA sequencing dataset, we hypothesize that their expression patterns may be subject to age-related effects specific to certain cell types. Such effects may only be identified through the use of single-cell analysis techniques.

### The relative abundance of OPCs in the adult SEZ declines in middle-age

To determine if the cellular composition of the SEZ was affected by age, we used generalized linear modeling to compare the proportion of each SEZ cluster between middle-age and youth. We did not detect differences in the relative abundance of any Astro/NSC cluster or neuroblasts between the two age groups (Extended Data Fig. 4-1), suggesting that the pool of potential NSCs remained stable during the investigated age range (16–53 years).

**Figure 4.**
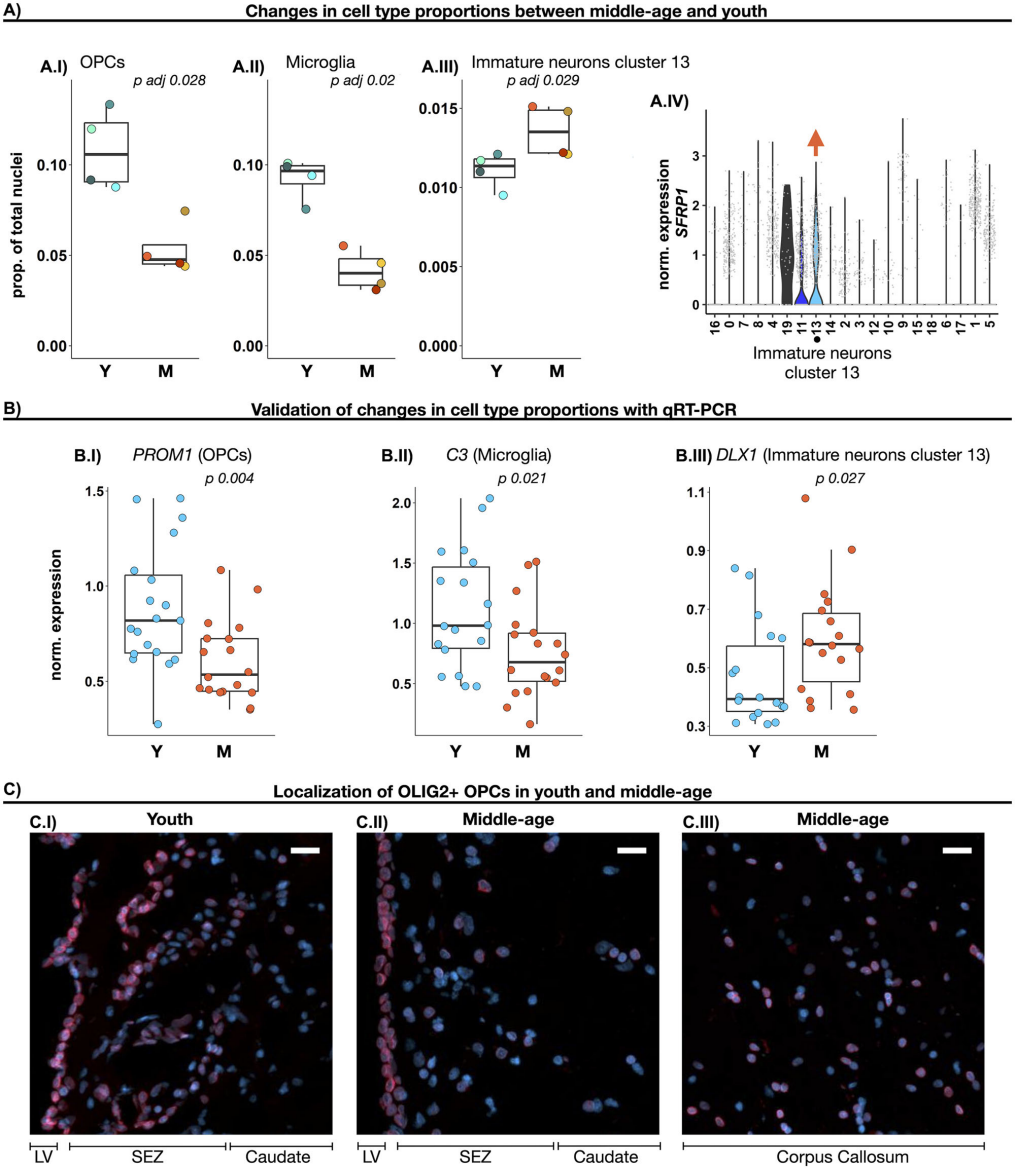
The relative abundance of SEZ OPCs, microglia, and immature neurons of cluster 13 differed between youth and middle-age. ***A***, Cell types in the SEZ with different relative abundance between middle-age (M) and youth (Y). ***A.I–A.III***, Box plots depicting the proportion of OPCs, microglia, and immature neurons (cluster 13) in Y and M. Each dot indicates a sample, and the horizontal lines indicate the median. Group comparison (Y vs M) was carried out with a generalized linear model and *p* values were Bonferroni corrected. ***A.IV***, Violin plots depicting *SFRP1* expression level in each cluster. Immature neurons of cluster 13, which are more abundant in M, highly express *SFRP1*. ***B***, Expression of marker genes for OPCs (*PROM1*), microglia (*C3*), and immature neurons cluster 13 (*DLX1*) in the SEZ of an independent cohort of 19 youth (average age = 20) and 19 middle-age cases (average age = 48). Normalized mRNA expression of *PROM1* (***B.I***), *C3* (***B.II***), and *DLX1* (***B.III***), obtained by qRT-PCR. Group comparison (Y vs M) was carried out with a *T* test (*PROM1* and *C3*) or Mann–Whitney *U* test (*DLX1*), depending on data distribution. ***C***, Representative images of OLIG2 + nuclei (red) in the SEZ of youth (***C.I***) and middle-age (***C.II***,***C.III***). All cases had abundant OLIG2 staining in the corpus callosum. All nuclei are stained with DAPI (blue). Scale bar = 20 μm; LV, lateral ventricle. Further supporting details for this figure are available in Extended Data Figures 4-1 and 4-2 and Extended Data Table 4-1.

The proportion of OPCs was reduced by ∼50% in middle-age as compared with youth (Fig. 4*A.I*). Additionally, the proportion of microglia was reduced in middle-age (Fig. 4*A.II*). In contrast, the proportion of the immature neuron cluster 13 was increased in middle-age (Fig. 4*A.III*). Among the immature neuron clusters, cluster 13 had the highest expression of *SFRP1* (Fig. 4*A.IV*), a Wnt antagonist that inhibits cell proliferation (Donega et al., 2022).

10.1523/ENEURO.0246-23.2024.f4-1 Figure 4-1 **Proportions of SEZ clusters in youth and middle-aged adults.** **A)** Boxplots depicting the proportion of all clusters in youth (Y) and middle-age (M). Each dot indicates a sample and horizontal lines indicate the median. **B)** Table depicting the results from comparing the relative abundance of each cluster between Y and M. Group comparison was carried out with generalized linear modeling and *p* values were Bonferroni corrected. Middle-age coef. is the value estimated for the coefficient associated with the middle-age variable in the regression model (effect of the middle-aged group on the probability that a nucleus belongs to a particular cluster). **C)** UMAP depicting nuclei Y M donors, colored by assigned cluster. The labels indicate clusters that changed their proportion in M as compared to Y. Download Figure 4-1, TIF file.

10.1523/ENEURO.0246-23.2024.f4-2 Figure 4-2 **Validation of changes in cell type proportions using bulk RNA sequencing data of the SEZ.** **A)** Box plots depicting the estimated proportion of OPCs, microglia and immature neurons cluster 13 in 4 different age groups. Proportions were estimated by deconvoluting bulk RNA sequencing data of the SEZ from 20 donors on the transcriptomic profiles of the SEZ clusters identified in snRNAseq. The average age of the donors in each group is indicated in the x axis. Group comparison for the relative abundance of OPCs, microglia and immature neurons cluster 13 was performed with Kruskal Wallis test, and a Dunn post hoc analysis was performed for OPCs. *p* values resulting from the Dunn Test were FDR corrected. * indicates adj. *p* value <0.05*.* **B)** Correlation between gene expression and age, for OPCs, microglia and immature neurons cluster 13 marker genes. **B.I)** Color-coded heatmap illustrating the correlation between gene expression activity and age in the bulk RNA sequencing dataset for marker genes for OPCs and microglia that exhibited negative correlation with age, and marker genes for immature neurons cluster 13 that exhibited positive correlation with age. Only top 5 marker genes per cluster are shown, based on fold change. Corresponding *p* values for each correlation are provided. **BII-BIII)** Scatter plots showing normalized expression activity in relation to age, for genes with possitive and negative correlation with age, respectively. Download Figure 4-2, TIF file.

10.1523/ENEURO.0246-23.2024.t4-1 Extended Data table 4-1 Spearman correlations between gene expression and age in a bulk RNA sequencing dataset of the human SEZ, for OPCs, microglia and immature neurons cluster 13 marker genes. Download Extended Data table 4-1, XLS file.

By deconvolving bulk RNA sequencing data from human SEZ samples (Extended Data Fig. 4-2A), we estimated the proportions of human SEZ clusters identified with snRNAseq. We observed a significant decrease in the relative abundance of OPCs in aged adults (average age = 86) with respect to young adults (average age = 23) and adolescents (average age = 16.5). In contrast, the relative abundance of microglia and cluster 13 immature neurons were not altered with age in the bulk RNAseq profiles. Nonetheless, this analysis has some limitations, particularly regarding its performance with “rare” cell types or similar cell states, such as the immature neuronal cluster 13. Additionally, it assumes that gene expression in bulk samples results from a linear combination of gene expression profiles from individual cell types. In reality, this can be more complex, potentially involving nonlinear interactions. Therefore, we examined the association between gene expression and age for marker genes for OPCs, microglia, and the immature neuronal cluster 13 in the bulk RNAseq data. We employed Spearman correlation to assess this association, as it can identify nonlinear relationships as well. We identified 55 OPCs and 37 microglia marker genes that negatively correlated with donors’ age (*p* value < 0.05) and 101 marker genes for cluster 13 that positively correlated with age (*p* value < 0.05; Extended Data Table 4-1). The top marker genes for each cluster, which also displayed a consistent change in expression activity in the bulk RNAseq data in accordance with the observed change in proportion in the snRNAseq dataset, are presented in Extended Data Figure 4-2B.

Additionally, we used qRT-PCR to evaluate the SEZ expression of marker genes for OPCs (*PROM1*; Beier et al., 2008; Choi et al., 2018; Marques et al., 2018; The Human Protein Atlas, 2024), microglia (*C3*; Guttikonda et al., 2021), and immature neuron cluster 13 (*DLX1*; Ghanem et al., 2007) as they are cluster marker genes and marker genes in the literature. qRT-PCR was performed in an independent cohort of 19 youth (mean age = 20 years) and 19 middle-aged cases (mean age = 48). *PROM1* and *C3* both had significantly reduced expression in middle-age compared with youth, whereas *DLX1* had significantly increased expression in middle-age (*p* value < 0.05; Fig. 4*B*). Altogether, snRNAseq, bulk transcriptomic, and qRT-PCR data suggested a reduction in OPCs and microglia proportion in the SEZ associated with age, while the proportion of immature neurons of cluster 13 seems to increase with the age of the donors.

Lastly, to confirm the presence of OPCs and determine their location in the human SEZ with a histological technique, we performed immunofluorescent staining of OLIG2 in the human SEZ from five youth and five middle-age cases. We detected OLIG2 + nuclei along the SEZ in youth and middle-age (Fig. 4*C.I*,*C.II*). In general, OLIG2 + cells appeared most dense in the corpus callosum (Fig. 4*C.III*) and then in the SEZ (Fig. 4*C.I*,*C.II*), and were detected at lower levels in the adjacent caudate nucleus (Fig. 4*C.I*,*C.II*).

## Discussion

In the present work, we provide evidence of ongoing neurogenesis in the human SEZ neurogenic niche of youth and adults. We found clusters of nuclei with transcriptional profiles consistent with most cell types found along the neuronal lineage from NSCs, neuroblasts, immature neurons, and mature neurons (likely adjacent to SEZ). We corroborated this by pseudotime analysis and by comparison with snRNAseq of the mouse SEZ. Transcripts related to nervous system development were reduced in middle-age as compared with youth, indicating that developmental processes decline in middle-age. Furthermore, we found the relative abundance of OPCs and microglia decline with age, in contrast to the higher proportion of neurons that highly express *SFRP1*, suggesting that the cellular composition of the human SEZ is remodeled with maturity.

**Figure 5.**
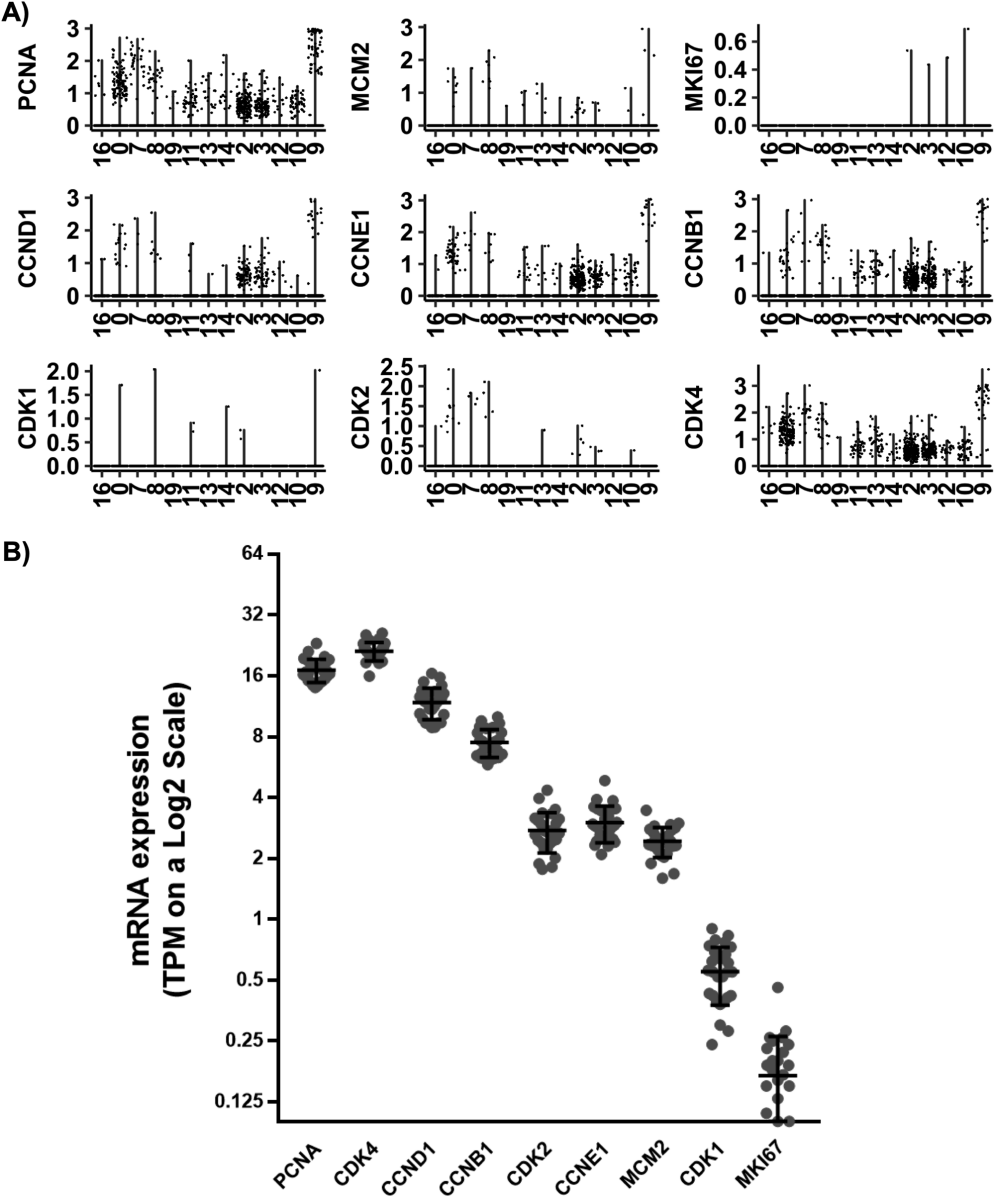
Expression of specific proliferation- and cell cycle progression–related genes. ***A***, While we identified cells consistent with all stages of neurogenesis, we did not find clusters significantly enriched for nine proliferation- or cell cycle progression–related genes (Donega et al., 2022). A small proportion of nuclei expressed PCNA, a gene widely accepted as a proliferation marker; however, its average expression level was highest in clusters 0, 7, and 9 corresponding with NSCs/Astro and neuroblasts. ***B***, We then quantified the expression of the nine proliferation genes from a previous bulk RNA sequencing of SEZ tissue from controls (*n* = 27, aged 21–66). The nine proliferation genes were detected, albeit with some at relatively low expression levels making them difficult to detect with snRNAseq sequencing depth.

The coexpression of niche astrocyte and NSC marker genes is consistent with studies suggesting NSCs have astrocytic-like origins in the adult human SEZ (Sanai et al., 2004). The low expression of some proliferation-related genes in the NSCs of the adult SEZ (Fig. 5) supports studies proposing neurogenesis as a relatively rare event in the adult human brain (Shimogori et al., 2004; Macas et al., 2006; Weissleder et al., 2016; Sorrells et al., 2018). However, we identified cell clusters highly expressing transcripts consistent with neuroblasts and immature neurons comprising ∼13% of SEZ nuclei. A possible explanation is that while stem cells seem to be mostly quiescent, a few active ones can produce significant numbers of neuronal precursors. Interestingly, human neuroblasts (cluster 9) were enriched in transcripts highly expressed by mouse neuroblasts and active NSCs (aNSCs; Zywitza et al., 2018) and were consistent with a high level of translation. Single-cell studies of the mouse brain found increased expression of mRNAs encoding proteins involved in transcription and translation in aNSCs (Llorens-Bobadilla et al., 2015; Zywitza et al., 2018; Xie et al., 2020), suggesting that translational activation is one of the earliest events when exiting quiescence (Llorens-Bobadilla et al., 2015). In addition, the neuroblast cell cluster we identified abundantly expressed *CCND2*, which promotes cell cycle progression and early neuronal differentiation and is essential for adult neurogenesis (Ekholm and Reed, 2000; Kowalczyk et al., 2004). We found that neuroblasts also express *CDC42*, which is critical for neural progenitor proliferation in the adult hippocampus via involvement with EGF and FGF (Vadodaria et al., 2013). *FGFR3* expression in the neuroblast cluster may indicate self-renewal of some neuroblasts (Maric et al., 2007), whereas the expression of *CEND1* could indicate other neuroblasts initiating cell cycle exit and early neuronal differentiation (Gaitanou et al., 2019). Taken together, we detected many cell cycle–related transcripts in the neuroblast cluster consistent with the ongoing proliferation of neuronal precursors in the adult human SEZ.

Pseudotime analysis identified *HSPA1A* expression as a potential driver of the transition between Astro/NSC and the neuroblast cluster, peaking in neuroblasts and decreasing in neurons. *HSPA1A* codes for a 70 kDa heat shock protein (Hsp70), and high *HSPA1A* expression activity has been associated with increased cancer cell proliferation (Boudesco et al., 2018), including in glioma (Zhao et al., 2021). The expression level of *HSPA1A* also modulates the cell cycle under physiological conditions and, therefore, may participate in the regulation of the transition between inactive and active NSC states. Future experiments tuning *HSPA1A* expression activity in mouse or human-derived NSCs could help to identify the possible role of *HSPA1A* in NSCs and neuroblasts. Our pseudotime trajectory is supported by evidence from single-cell transcriptomics in the mouse hippocampus that found markers distinguishing stages of neural progenitor cells into early stage (*CCND2*, *HMGN2*) and later stage progenitors (*DCX*; Artegiani et al., 2017), which we also find in immature neurons. This supports that similar stages of neurogenic cells can be found in the adult human SEZ.

We did not identify changes in the relative abundance of Astro/NSCs or neuroblasts between the youth and middle-aged groups, suggesting that the potential for SEZ neurogenesis does not decline significantly in adulthood. Our findings are in contrast to some previous human studies claiming that progenitor cells in the SEZ decrease to negligible levels in adulthood (Sanai et al., 2011; Bergmann et al., 2012; Coletti et al., 2018). One possible reason for this is that we have surveyed a larger amount of SEZ tissue than what was examined in earlier studies. While the abundance of Astro/NSCs remained stable, we found lower levels of transcripts related to nervous system development in Astro/NSCs and ependymal and neuronal nuclei in middle-age, which suggests less plasticity all along the neuronal trajectory. Indeed, the SEZ qNSCs from older mice are more resistant to injury-induced activation than NSCs from young mice (Kalamakis et al., 2019). In addition, we identified higher expression of genes related to immune activation in oligodendrocytes in middle-age. Bulk transcriptomic data also indicated further immune activation associated with later stages of aging in the human SEZ (Bitar et al., 2022). Inflammatory signals derived from this cellular niche promote quiescence of NSCs in the mouse SEZ (Solano Fonseca et al., 2016; Kalamakis et al., 2019) and potentially in humans with elevated inflammation, such as in schizophrenia patients (North et al., 2021). Our results suggest that mature oligodendrocytes are contributing to the increase in proinflammatory signals associated with age in the human SEZ and could decrease the probability of qNSCs reentering the cell cycle during middle adulthood.

The relative abundance of OPCs declined in middle-age as determined by a reduction in the proportion of the OPC cluster and further validated with bulk RNA sequencing data and qRT-PCR from an independent SEZ cohort. The SEZ harbors a large pool of OPCs compared with the adjacent caudate nucleus, making it an important region for oligodendrocyte production throughout life. There is an age-related reduction in the remyelination potential of the CNS. This is reflected by reductions in white matter volume across different brain regions in aging (Liu et al., 2016) and is associated with cognitive decline (Coelho et al., 2021). In disease, patients with multiple sclerosis frequently transit from a relapsing-remitting to a progressive form of the disease ∼45 years of age, with irreversible worsening of lesions and neurologic function (Tutuncu et al., 2012). Interestingly, the SEZ from patients with multiple sclerosis contains an increased number of proliferating OPCs, suggesting that the human SEZ contributes OPCs that may be available to migrate to white matter lesions to aid remyelination (Nait-Oumesmar et al., 2007). However, the lower proportion of endogenous SEZ OPCs in middle adulthood might constitute a limitation that could contribute to the age-related decline in CNS remyelination efficiency in normal aging, and potentially in MS.

The relative abundance of microglia was also decreased in middle-age as compared with youth. Elevated states of inflammation in the SEZ are associated with reduced expression of key microglia markers in schizophrenia (North et al., 2021, 2022). A decline in the proportion of SEZ microglia between early and middle adulthood, coinciding with elevated immune transcripts in mature oligodendrocytes might constitute a hallmark of early age-related changes in the SEZ, where inflammation is further elevated during aging (Galatro et al., 2017; Olah et al., 2018; Sankowski et al., 2019). Conversely, the proportion of immature neuron cluster 13, which highly expressed *DLX6-AS1* and *EGFR*, together with *SFRP1*, was greater in middle-aged donors. Donega et al. (2022) demonstrated that an increase in the number of SFRP1-positive cells in the human dorsal SEZ is associated with later stages of aging and proposed that reducing SFRP1 may reactivate neuronal progenitor cells. Thus, the higher relative abundance of immature neurons expressing *SFRP1* (cluster 13) in middle-age could reflect a reduction in their proliferative potential. Alternatively, an increase in immature neurons in middle-age may indicate deficits in their differentiation and migration out of the SEZ.

Many transcript levels differed between youth and middle-age across multiple cell types and may indicate a transcriptional hallmark of age that affects several cell types. For instance, MSPNs, Astro/NSCs, and ependymal cells all depicted lower *CPE* expression levels in middle-age. Lower *CPE* expression is associated with learning and memory decline in mice (Woronowicz et al., 2008; Li et al., 2021), and low *CPE* expression in middle adulthood might contribute to cognitive changes in later adult years in humans (Wisdom et al., 2012). In addition, the expression of *MAN2A1* was lower in immature neurons and MSPNs in middle-age. A previous study reported a positive association between *MAN2A1* expression and global cognitive scores (Fabbri et al., 2022); therefore, low *MAN2A1* expression in middle-aged donors may also be related to reduced cognitive flexibility associated with age. While *SLC6A1-AS1* expression was increased in immature neurons and MSPNs in middle-age, high *SLC6A1-AS1* expression predicts better survival from cancer (cholangiocarcinoma; Wang et al., 2018), suggesting a potential inhibitory effect of SLC6A1-AS1 on cellular proliferation. Thus, the high neural expression of *SLC6A1-AS1* may signal reduced proliferative potential in middle-age. On the other hand, some changes in gene expression between early and middle adulthood may reflect protective mechanisms against age-related brain damage, such as axonal degeneration (Salvadores et al., 2017). For instance, the expression of *SEMA6D*, an axon guidance molecule (Ueno et al., 2020), was lower in OPCs, Astro/NSCs, and MSPNs in middle-age as compared with youth. Increased SEMA6D expression acts as an axon repellent that impairs axonal regrowth after injury (Ueno et al., 2020). Therefore, the reduced expression of *SEMA6D* in middle-age might reflect a buffering mechanism for axonal repair during normal aging.

Some limitations may be considered when interpreting the results presented in this study. First, we were not able to discriminate between astrocytes and NSCs. As mentioned above, it may be that astrocytes constitute the NSCs in the adult human SEZ (Sanai et al., 2004). However, the lack of knowledge regarding specific markers to distinguish these cell types (Lim and Alvarez-Buylla, 2016) could also explain our inability to resolve them. Also, we failed to detect a separate cluster of actively proliferating NSCs with snRNAseq for a number of possible reasons (Fig. 5*A*). Firstly, cell proliferation is a transient process meaning there are few *Ki67* + and/or *PCNA* + nuclei at a given time point [∼5 Ki67-positive cells/50 μm of SEZ (Weissleder et al., 2016)], and the transcription factors regulating proliferation are expressed at relatively low abundance (Fig. 5*B*). Additionally, a recent review has emphasized the specific difficulty encountered by snRNAseq when attempting to detect the expression of proliferation markers, even in highly proliferative tissues (Tosoni et al., 2023). Therefore, we may lack sensitivity with the limited number of nuclei per sample and sequencing depth per nuclei. Secondly, proliferating cells undergo vast nucleic changes including doubling of DNA and breakdown of the nuclear envelope which could result in exclusion or loss during nucleic isolation and fluorescence-activated sorting. This may preclude the identification of a NSC cluster in an active cycle, limiting our ability to delve into potential differences in NSC proliferation between early and middle adulthood. Lastly, considering the potential implications of the observed decrease in OPC abundance in the SEZ associated with age, it might be pertinent to validate this using additional OPC marker genes. While *PROM1* has been identified as an OPC marker in prior brain scRNAseq studies (Marques et al., 2018; The Human Protein Atlas, 2024) and its expression aligns with the OPC cluster in our data, there is evidence suggesting that PROM1+ generates oligodendrocytes in a nonspecific manner (Wang et al., 2013).

In summary, different cell types in the adult human SEZ form a trajectory from NSCs and neuroblasts to mature neurons, and their relative abundance appears stable between youth and middle-aged adults. Nevertheless, age affects the transcriptomic profile of several adult cell types in the SEZ, particularly reducing the expression of genes related to nervous system development. In addition, the relative abundance of OPCs and microglia decreases with age, probably reducing the remyelination efficiency of the CNS. The transcriptional changes in oligodendrocytes may lead to altered inflammatory phenotypes later in life. Our results identify the cell types and genes particularly changed from youth to middle adulthood in the human SEZ and may help identify key targets to modulate or increase the neurogenic capacity of this niche.

## Data Availability Statement

All gene expression data in the presented manuscript are available through the GEO of NCBI, accession number GSE234790. The code used for analysis is publicly available at https://github.com/sofiapuvogelvittini/humanSEZ_snRNAseq_analysis. Single-nucleus RNAseq characterizes the cell types along the neuronal lineage in the adult human subependymal zone and reveals reduced oligodendrocyte progenitor abundance with age.

## Synthesis

Reviewing Editor: Matthew Grubb, King's College London Decisions are customarily a result of the Reviewing Editor and the peer reviewers coming together and discussing their recommendations until a consensus is reached. When revisions are invited, a fact-based synthesis statement explaining their decision and outlining what is needed to prepare a revision will be listed below. The following reviewer(s) agreed to reveal their identity: Sara Zeppillli.

Both reviewers are positive about this revised manuscript. They suggest some important but relatively straightforward further revisions that are necessary to ensure the results fully support the study's conclusions. Please address all of these.

Reviewer 1:

Generally, I am satisfied with the changes made to the manuscript and I believe that it is improved overall. I would, however, suggest some more explanation for the choice of Prom1 as a marker to validate for OPCs. I can see this is one of the predicted markers for OPCs in Figure 1C, however, this would be viewed as an uncommon marker for OPCs.

Reviewer 2:

In this study, the authors conducted a comprehensive investigation using single-nucleus transcriptomics of the adult human SVZ across two distinct age groups: young individuals (aged 16-22) and middle-aged individuals (aged 44-53).

The two primary objectives of this study can be summarized as follows:

i) The first objective was to systematically characterize the cellular composition and the transcriptional landscape of the adult human lateral/ventral SVZ. This study addresses the ongoing debate regarding the presence of neural stem cells and active progenitors in the human SVZ. To infer cellular identities, the authors primarily used established marker genes combined with differential gene expression analysis. In addition, the authors also compared their human dataset with adult mouse SVZ datasets. Finally, they used pseudotime trajectory analysis to infer potential differentiation trajectories among these cellular identities.

ii) The second objective was to identify transcriptomic differences between the two age groups, namely young and middle-aged donors. This was accomplished by comparing gene expression and the relative cellular abundance between young and middle-aged individuals.

The findings of this study reveal molecular signatures indicative of potentially active neural stem cells and neuroblasts positioned along a differentiation trajectory within the adult human SVZ. This provides support for the notion that, while reduced compared to earlier developmental stages and compared to mice, neurogenesis continues to be an active cellular process also in the SVZ of humans. Furthermore, the findings of this study reveal transcriptomic differences between young and middle-aged groups. These differences suggest the retention of developmental signatures in young donors compared to later stages, and the presence of dynamic cellular and molecular reconfigurations during the aging process.

The authors strengthened their observations through resubmission, reinforcing experimentally and computationally the key finding of the alteration in cellular composition during aging. Additionally, they used an alternative computational well-established tool, Monocle3, to support the inference of cellular differentiation trajectories.

In conclusion, this study enhances our comprehension of the cellular and molecular processes in the adult human brain, and holds implications for the advancement of our understanding of psychiatric and neurological disorders. The selection of donor age groups was also critical, as it captures different dynamic and plastic processes in the human brain. Nevertheless, I suggest that the authors further support one of the key findings of the study - the differentiation trajectory of neural stem cells/astrocytes towards neurons and not glia - with additional computational analysis.

Main point:

The use of an alternative pseudotime trajectory analysis across all cell types of the SEZ - Monocle3 - was valuable to further support the differentiation trajectory of neural stem cells/astrocytes towards neurons. However, while pseudotime trajectory tools provide valuable insights, they also come with limitations, and offer only a partial perspective on biological interpretation, particularly when the results lack experimental validation. Unexpectedly, the alternative analysis conducted by the authors using Monocle3, including all cells, revealed molecular similarities between OPCs and immature neurons, rather than between OPC and oligodendrocytes. This result introduces ambiguity into the interpretation of the key finding on the neuronal lineage. Therefore, I strongly recommend the authors to repeat the pseudotime trajectory analysis, by including all cells, also with their primary pseudotime trajectory tool, destiny. This additional analysis will, at the very least, provide insight into whether the unexpected outcome stems from the inherent limitations of these tools, or if there are some shared cellular and molecular features between these two unrelated cell types that warrant consideration in future investigations. Additionally, I wonder whether the authors accounted for cell cycle-related genes through regression, as this could influence the interpretation of the trajectories between cell identities and cell states. Unfortunately, this detail was not clear in the method section.

Minor points:

1) I suggest that the authors explicitly state at the beginning of the results section that age groups were pooled and analyzed together.

2) I recommend the authors to add cell type identity labels in the UMAP plots of the Extended Data - Figure 2-1, and to specify in the caption what the numbers are.

3) I recommend that the authors include, in the Extended Data section, the number of genes per nucleus across all cell types that were sequenced, not just across samples, as it appears that any QC filtering was performed on the genes/nucleus beyond Cell Ranger's default procedure. This information will help to assessing potential variability across cell types, and to understanding whether specific cell types were more impacted than others during the experimental and computational steps.

4) The Github link to their code at the end of the method section does not function, and thus could not be checked whether is available, likely for keeping privacy of the authors?

## Author Response

snRNAseq SEZ revision2:

We would like to thank the reviewers for their constructive input. We aimed to address all of their suggestions.

Reviewer 1:

1) Generally, I am satisfied with the changes made to the manuscript and I believe that it is improved overall. I would, however, suggest some more explanation for the choice of Prom1 as a marker to validate for OPCs. I can see this is one of the predicted markers for OPCs in Figure 1C, however, this would be viewed as an uncommon marker for OPCs.

We chose PROM1 due to its predominant expression within the OPCs cluster, as indicated by our transcriptional data. This observation aligns with findings from previous single-cell RNA sequencing studies conducted on mouse [1] and human brain [2]. In addition, Prom1 has been proposed as a marker of "stemness" in high-grade oligodendroglial tumors [3]. We have now included these references on page 23. However, it is essential to acknowledge a potential limitation highlighted in the literature, indicating that PROM1+ cells may generate oligodendrocytes in a non-specific manner [4]. We have incorporated this information as a limitation to our study, on page 29, and emphasized the importance of future validations. To strengthen our findings regarding the age-associated decrease in OPCs within the SEZ, we recommend conducting additional experiments using a combination of different OPC marker genes. This approach will provide a more comprehensive and robust validation of our observations.

Reviewer 2:

1) The use of an alternative pseudotime trajectory analysis across all cell types of the SEZ - Monocle3 - was valuable to further support the differentiation trajectory of neural stem cells/astrocytes towards neurons. However, while pseudotime trajectory tools provide valuable insights, they also come with limitations, and offer only a partial perspective on biological interpretation, particularly when the results lack experimental validation. Unexpectedly, the alternative analysis conducted by the authors using Monocle3, including all cells, revealed molecular similarities between OPCs and immature neurons, rather than between OPC and oligodendrocytes. This result introduces ambiguity into the interpretation of the key finding on the neuronal lineage. Therefore, I strongly recommend the authors to repeat the pseudotime trajectory analysis, by including all cells, also with their primary pseudotime trajectory tool, destiny. This additional analysis will, at the very least, provide insight into whether the unexpected outcome stems from the inherent limitations of these tools, or if there are some shared cellular and molecular features between these two unrelated cell types that warrant consideration in future investigations.

We have incorporated pseudotime analysis using Destiny, encompassing all cell types. The result is presented in Figure 2-2 and discussed on page 19.

Figure 2-2. Additional pseudotime analysis with Destiny.

Trajectory graph inferred with Destiny, including all cell types and nuclei. A) Nuclei are ordered in a three-dimensional diffusion map (DC=diffusion components). Colors indicate original nuclei clusters. A.I-A.II) Present the identical graphs from different perspectives. B) Pseudotime (PDT) estimation, indicated in a color code, calculated based on the diffusion components. The origin of the pseudotime trajectory was automatically identified. OPCs situated in proximity to immature neurons, consistent with findings from Monocle3 analysis.

2) Additionally, I wonder whether the authors accounted for cell cycle-related genes through regression, as this could influence the interpretation of the trajectories between cell identities and cell states. Unfortunately, this detail was not clear in the method section." We did not regress on cell-cycle related genes because if there were differences in the expression of these genes between cell types or age groups, we were keen to identify them. We have know stated this in materials and methods (page 8) 3) I suggest that the authors explicitly state at the beginning of the results section that age groups were pooled and analyzed together.

We have included this suggestion at the beginning of the results section (page 14).

4) I recommend the authors to add cell type identity labels in the UMAP plots of the Extended Data - Figure 2-1, and to specify in the caption what the numbers are.

We have now specified this in the legend of Figure 2-1.

5) I recommend that the authors include, in the Extended Data section, the number of genes per nucleus across all cell types that were sequenced, not just across samples, as it appears that any QC filtering was performed on the genes/nucleus beyond Cell Ranger's default procedure. This information will help to assessing potential variability across cell types, and to understanding whether specific cell types were more impacted than others during the experimental and computational steps.Include the number of genes per nucleus across all cell types that were sequenced.

This has been addressed with Extended Data table 1-4.

6) The Github link to their code at the end of the method section does not function, and thus could not be checked whether is available, likely for keeping privacy of the authors?:

Certainly, the double-blinded review process of the journal required this discretion. The complete GitHub link will be made available upon the publication of the manuscript.

1. Marques, S., D. van Bruggen, D.P. Vanichkina, et al., Transcriptional Convergence of Oligodendrocyte Lineage Progenitors during Development. Dev Cell, 2018. 46(4): p. 504-517.e7.

2. ATLAS, T.H.P. THE HUMAN PROTEIN ATLAS, Available from: https://www.proteinatlas.org/ENSG00000007062-PROM1/single+cell+type/brain [Accessed 01 January 2024]. 2024.

3. Beier, D., J. Wischhusen, W. Dietmaier, et al., CD133 Expression and Cancer Stem Cells Predict Prognosis in High-grade Oligodendroglial Tumors. Brain Pathology, 2008. 18(3): p. 370-377.

4. Wang, J., M.A. O'Bara, S.U. Pol, et al., CD133/CD140a-Based Isolation of Distinct Human Multipotent Neural Progenitor Cells and Oligodendrocyte Progenitor Cells. Stem Cells and Development, 2013. 22(15): p. 2121-2131.
