# Supplementary figures and images for: Single-Nucleus RNA-Seq Characterizes the Cell Types Along the Neuronal Lineage in the Adult Human Subependymal Zone and Reveals Reduced Oligodendrocyte Progenitor Abundance with Age
Source: eNeuro. 2024 Mar 1;11(3):ENEURO.0246-23.2024. doi: 10.1523/ENEURO.0246-23.2024 (PMC10913050; doi:10.1523/ENEURO.0246-23.2024)

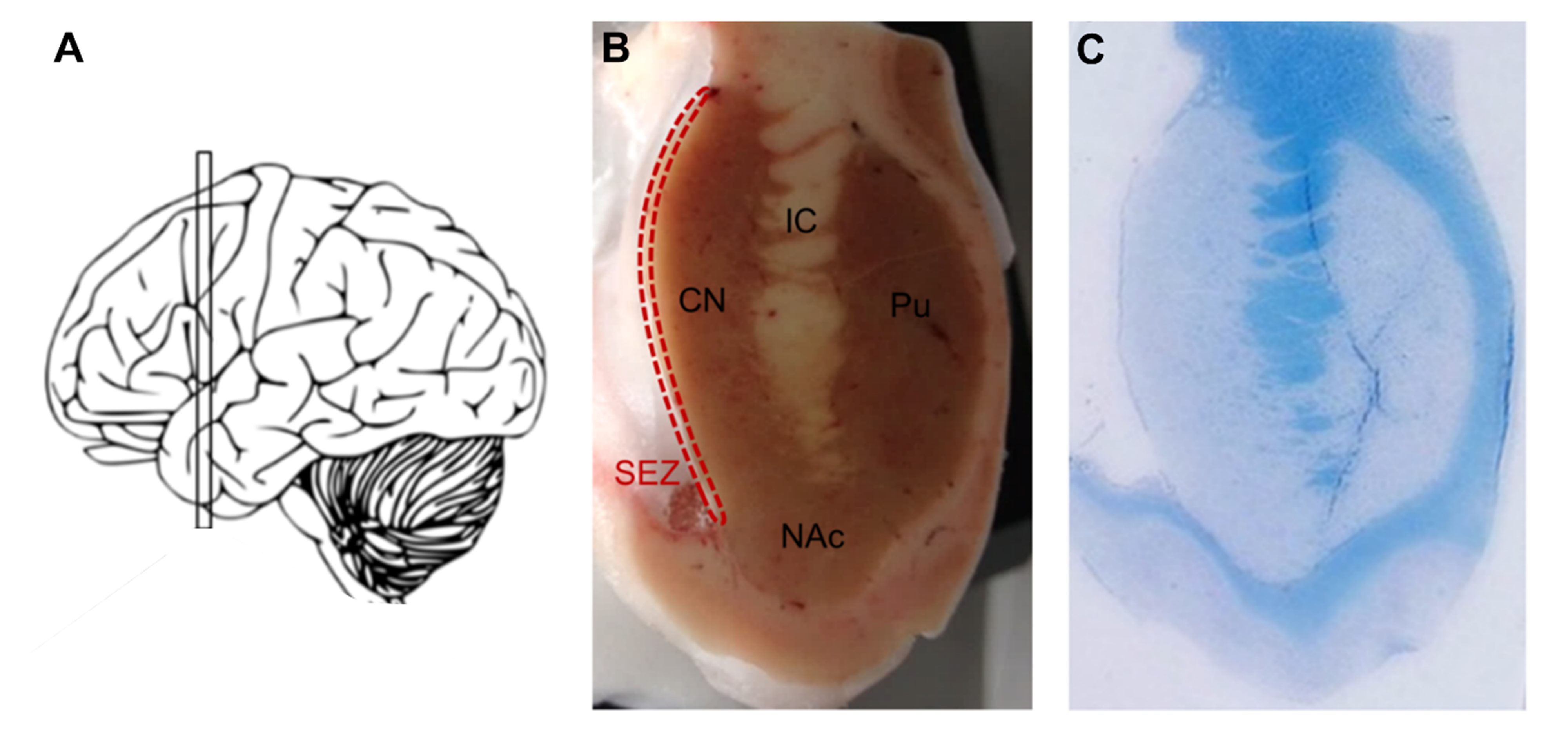

Supplement: Figure 1-1 — Fresh-frozen tissue cutting and dissection of the SEZ. A) Tissue from the rostral third of the basal ganglia was sectioned in the coronal plane into 7 × 100 μm sections. B) The SEZ (red dashed line) was dissected ∼2 mm deep to the surface of the lateral ventricle to include dorsal, middle and ventral regions. C) Luxol fast blue staining for myelin depicts the internal capsule separating the caudate nucleus and putamen as well as the external capsule surrounding the striatum. CN, caudate nucleus; IC, internal capsule; NAc, nucleus accumbens; SEZ, subependymal zone; Pu, putamen. Download Figure 1-1, TIFF file. [file eneuro-11-ENEURO.0246-23.2024-s002.tif]

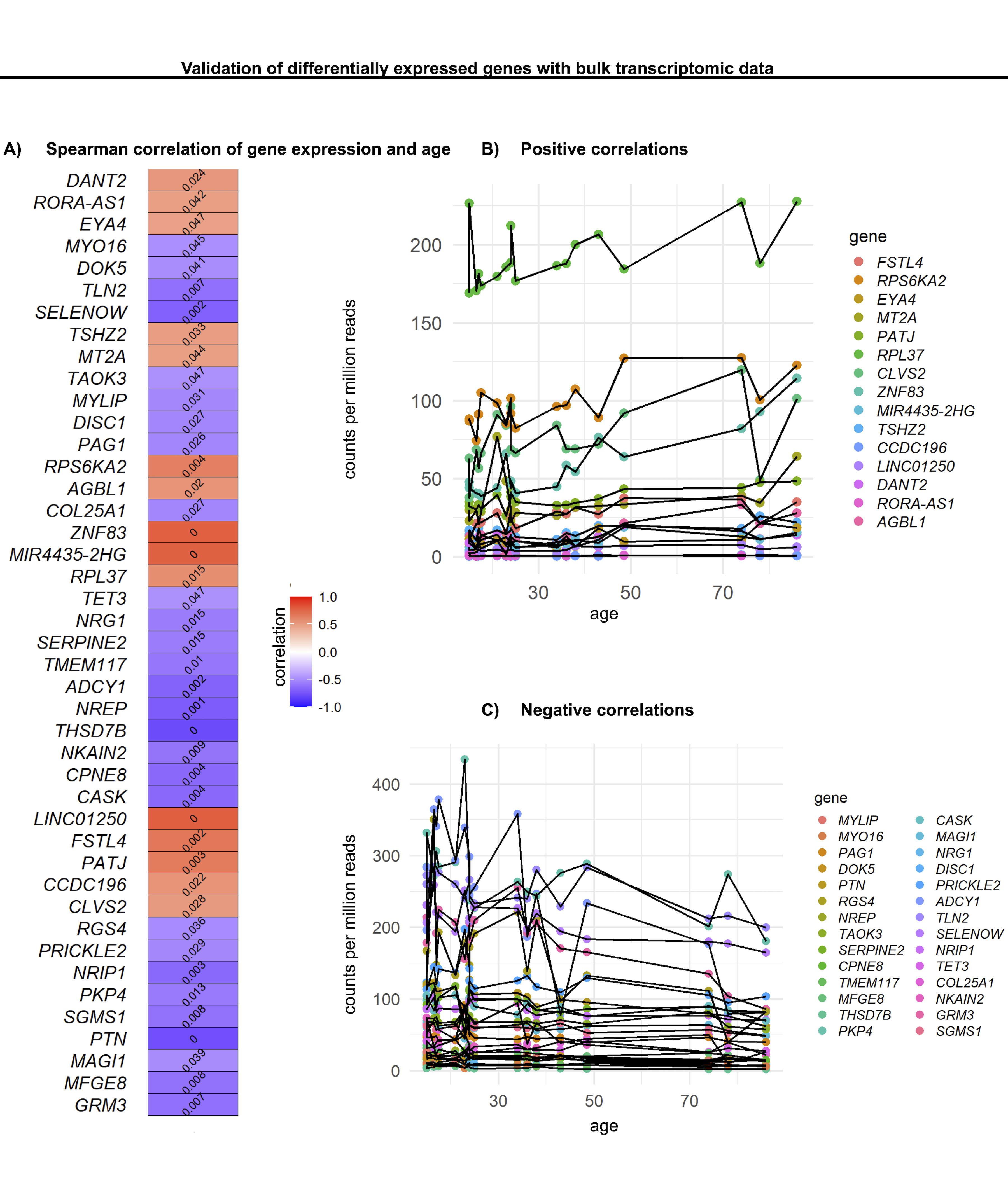

Supplement: Figure 3-3 — Validation of differentially expressed genes with bulk transcriptomic data. A) Heatmap depicting differentially expressed genes between youth and middle-age for which its expression activity was also significantly correlated with age in a bulk RNA sequencing dataset of the SEZ, collected from 20 donors spanning an age range of 15 to 86 years. The correlation between gene expression activity and age is shown in color code, for genes that shown a consistent change associated with age in both the snRNA and the bulk sequencing dataset. Corresponding p values for each correlation are provided. B-C) Scatter plots showing normalized expression activity in relation to age, for genes with possitive and negative correlation with age, respectively. Download Figure 3-3, TIF file. [file eneuro-11-ENEURO.0246-23.2024-s007.tif]

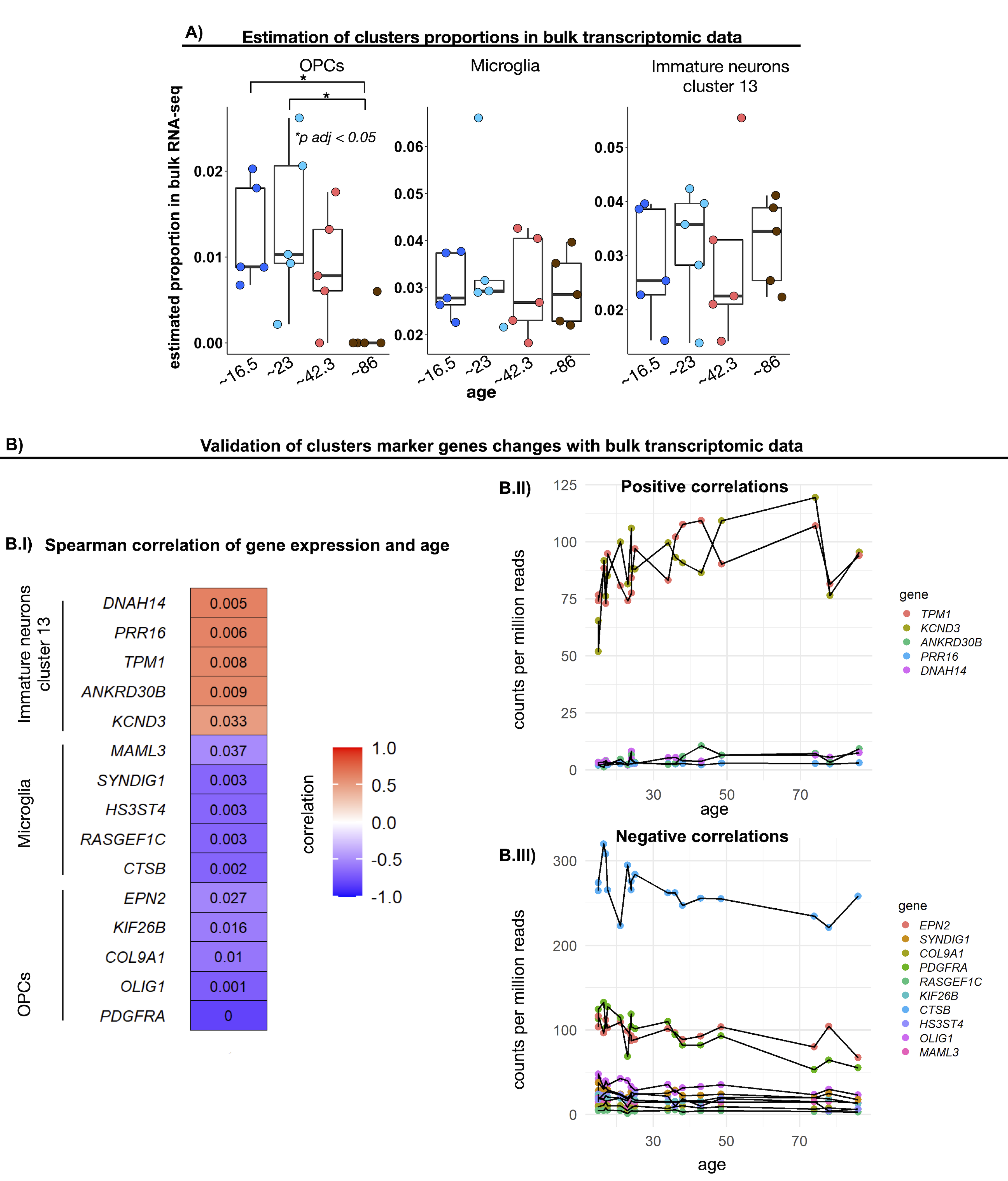

Supplement: Figure 4-2 — Validation of changes in cell type proportions using bulk RNA sequencing data of the SEZ. A) Box plots depicting the estimated proportion of OPCs, microglia and immature neurons cluster 13 in 4 different age groups. Proportions were estimated by deconvoluting bulk RNA sequencing data of the SEZ from 20 donors on the transcriptomic profiles of the SEZ clusters identified in snRNAseq. The average age of the donors in each group is indicated in the x axis. Group comparison for the relative abundance of OPCs, microglia and immature neurons cluster 13 was performed with Kruskal Wallis test, and a Dunn post hoc analysis was performed for OPCs. p values resulting from the Dunn Test were FDR corrected. * indicates adj. p value <0.05. B) Correlation between gene expression and age, for OPCs, microglia and immature neurons cluster 13 marker genes. B.I) Color-coded heatmap illustrating the correlation between gene expression activity and age in the bulk RNA sequencing dataset for marker genes for OPCs and microglia that exhibited negative correlation with age, and marker genes for immature neurons cluster 13 that exhibited positive correlation with age. Only top 5 marker genes per cluster are shown, based on fold change. Corresponding p values for each correlation are provided. BII-BIII) Scatter plots showing normalized expression activity in relation to age, for genes with possitive and negative correlation with age, respectively. Download Figure 4-2, TIF file. [file eneuro-11-ENEURO.0246-23.2024-s009.tif]
